# Reverse engineering gene regulatory networks from measurement with missing values

**DOI:** 10.1186/s13637-016-0055-8

**Published:** 2017-01-10

**Authors:** Oyetunji E. Ogundijo, Abdulkadir Elmas, Xiaodong Wang

**Affiliations:** Department of Electrical Engineering, Columbia University, 500 W 120th Street, New York, 10027 NY USA

**Keywords:** Gene expression, Missing data, Bayesian inference, Gaussian filters, Network inference

## Abstract

**Background:**

Gene expression time series data are usually in the form of high-dimensional arrays. Unfortunately, the data may sometimes contain missing values: for either the expression values of some genes at some time points or the entire expression values of a single time point or some sets of consecutive time points. This significantly affects the performance of many algorithms for gene expression analysis that take as an input, the complete matrix of gene expression measurement. For instance, previous works have shown that gene regulatory interactions can be estimated from the complete matrix of gene expression measurement. Yet, till date, few algorithms have been proposed for the inference of gene regulatory network from gene expression data with missing values.

**Results:**

We describe a nonlinear dynamic stochastic model for the evolution of gene expression. The model captures the structural, dynamical, and the nonlinear natures of the underlying biomolecular systems. We present point-based Gaussian approximation (PBGA) filters for joint state and parameter estimation of the system with *one-step* or *two-step missing measurements*. The PBGA filters use Gaussian approximation and various quadrature rules, such as the unscented transform (UT), the third-degree cubature rule and the central difference rule for computing the related posteriors. The proposed algorithm is evaluated with satisfying results for synthetic networks, in silico networks released as a part of the DREAM project, and the real biological network, the in vivo reverse engineering and modeling assessment (IRMA) network of yeast *Saccharomyces cerevisiae*.

**Conclusion:**

PBGA filters are proposed to elucidate the underlying gene regulatory network (GRN) from time series gene expression data that contain missing values. In our state-space model, we proposed a measurement model that incorporates the effect of the missing data points into the sequential algorithm. This approach produces a better inference of the model parameters and hence, more accurate prediction of the underlying GRN compared to when using the conventional Gaussian approximation (GA) filters ignoring the missing data points.

**Electronic supplementary material:**

The online version of this article (doi:10.1186/s13637-016-0055-8) contains supplementary material, which is available to authorized users.

## Introduction

Gene regulation happens to be one of the most important processes that take place in living cells [1, 2]. For instance, it includes controls over the transcription of messenger RNA (mRNA) and the eventual translation of mRNA into protein via gene regulatory networks (GRNs). A detailed network may depict various inter-dependencies among genes where nodes of the network represent the genes and the edges correspond to interactions among the genes [3]. The strength of these interactions represents the extent to which a gene is affected by other genes in the network. For instance, some of the genes encode specific proteins, known as the transcription factors that can bind deoxyribonucleic acid (DNA) as part of a complex or independently and regulate their rate of transcription [4, 5]. Binding of the DNA by the transcription factors may, in some occasions, include genes that encode for other transcription factors and also genes that encode proteins for other functions. Hence, this results in a complex level of interaction among the genes in the cell. Among others, understanding the complex intracellular network in a human cell may lead to the identification of diseased genes, drug targets, and biomarkers for complex diseases [6]. Thus, identifying the structure of GRNs has become a major focus in the systems approach to biology [7–10].

The generation of high throughput time series measurement of transcript levels (e.g., via microarray experiments) has become an increasingly powerful tool for investigating complex biological processes and a useful resource for GRN inference [11]. Modeling of the gene networks with gene expression data can be loosely categorized into static and dynamic models. A static approach to modeling gene expressions makes use of the following properties: correlation, statistical independence for clustering, and mutual information [12, 13]. Particularly, the clustering approach has gained significant popularity [14, 15]. On the other hand, the dynamic modeling of GRNs from time series data has also received considerable interest. For instance, Boolean network models quantize the empirical gene expression data into binary values [16] and view the network structures as constraints. Further, via the estimation of the parameters in S-systems, a kind of nonlinear mathematical models based on power law, few authors like [17, 18] have performed the reverse engineering of GRNs. Probabilistic Boolean network models are an extension to the Boolean network models which incorporate the inherent stochasticity of gene expression and the uncertainties introduced by the measurement noise [19]. Also, dynamic Bayesian networks (DBNs) have been proposed to model the time series gene expression data [20, 21] because DBNs can model stochasticity and handle noisy/hidden variables.

The state-space approach, an extension of the DBNs, is a popular technique to model the GRNs [22, 23], where the hidden state of the network can be estimated by Gaussian approximation (GA) filters. The conventional Kalman filter, being optimal for a linear Gaussian system [24], requires some modifications to be able to cope with the nonlinearity of the activation function that regulates the gene activity profile. For instance, the extended Kalman filter (EKF) uses the first-order terms of the Taylor’s series expansion [25] to linearize the nonlinear functions in the model. The EKF only calculates the posterior densities accurately to the first order with all higher moments truncated. A different paradigm of the GA filtering approach is the point-based filtering technique, which involves numerically integrating nonlinear functions by using a set of deterministic points. This approach lowers the computational complexity when compared to the Monte Carlo numerical integration which relies on randomly generated points, since it requires much less number of points with the same accuracy.

However, in reality, gene expression time series data may not contain sufficient quantity of data in the appropriate format for the inference of GRNs because of the missing data points [26]. For example, in microarray measurement of gene expression, errors such as insufficient resolution and image corruption or simply due to dust or scratches on the slide of a microarray chip may occur in the experimental process which lead to corruption or absence of some expression measurements. In the engineering literature, similar problems are inherent in networked control systems (NCS) and sensor networks where packet dropouts and time delays are an unavoidable phenomenon during data transmission [27]. Classical methods fail to solve the filtering and estimation problems for such cases with delays and missing data and cannot accurately infer the underlying network structure.

In this paper, we present a class of GA filters for inferring GRN from data with missing measurement values, which can be modeled in the same unifying framework as in the case of state estimation from one-step or two-step randomly delayed measurements [28]. A general framework is presented through augmenting the state variables and with Gaussian assumptions on the posterior state and missing measurement. To make GRN inference from measurements that contain missing data, we describe the network by a nonlinear model and a measurement model that incorporates the missing data. The inferred parameter set can be used to identify the underlying regulatory network structure.

In the literature, several point-based Gaussian approximation (PBGA) filters have been used for solving the GRN inference problem from DNA microarray gene expression data and genome-wide knockout fitness data [29, 30]; however, there is no solution that outperforms all other counterparts. Thus, one has to pick the filter balancing the estimation performance, implementation complexity, and filter stability. Prominent among the PBGA filters are the cubature Kalman filter (CKF) that makes use of the third-degree cubature rule [31], the unscented Kalman filter (UKF) that makes use of the unscented transformation [30, 32], and the central difference Kalman filter (CDKF) that makes use of the difference rule.

The remainder of this paper is organized as follows. In Section 2, we describe the system model and problem formulation. In Section 3, we describe the corresponding GA filter. In Section 4, we investigate the performance of the proposed algorithm on a synthetic network and a diverse set of in silico networks released as a part of the DREAM project, from which observations can be made for benchmarking purposes [33, 34]. In addition, we present results on a real data obtained from the IRMA network of yeast *Saccaromyces cerevisiae* [35]. Finally, Section 5 concludes the paper.

In this paper, we use the following notations:

1. $\mathcal {N}(\mathrm {x};\mu, \Sigma)$ denotes the Gaussian probability density function with mean *μ* and covariance *Σ*.
2. $\mathbb {E}_{g}\lbrace \cdot \vert \mu, \Sigma \rbrace $ denotes the Gaussian integral with respect to $\mathcal {N}(\mathrm {x};\mu, \Sigma)$.
3. $\hat {\mathrm {x}}$ represents the estimate of variable x, $\tilde {\mathrm {x}}= \mathrm {x}-\hat {\mathrm {x}}$ is the estimation error, and $\mathbb {E}[\!\cdot ]$ denotes the expectation operation.
4. **X** ^−1^ and **X** ^*T*^ represent the inverse and transpose of matrix **X**, respectively, and *I*_*n*_ denotes the *n*-dimensional identity matrix.

## Methods

### Problem formulation and system model

Gene regulatory networks can be modeled as either static or dynamic systems. In this paper, the state-space model is used which is an instance of the dynamic modeling and can effectively cope with time variations in the gene expression data. Consider a GRN consisting of *N* genes. Let *g*_*i,k*_,*i*=1,…,*N,k*=1,…,*K* denote the gene expression level for the *i*th gene at time step *k* where *K* is the total number of data points available. Here, “time” is a discrete index enumerating data points sampled at regular intervals. A well-adopted nonlinear model [25, 30] that captures the gene interactions and the evolution of gene expression values effectively is the discrete-time nonlinear stochastic dynamical system which is proposed in [36] as follows:

1 $$ \begin{aligned} g_{k,i} = \sum_{j=1}^{N} a_{ij}g_{k-1,j} + \sum_{j=1}^{N} b_{ij}\,f(g_{k-1,j},\mu_{j})+ I_{0i} + e_{k-1,i}\\ i,j = 1,\ldots,N, \quad k = 1,\ldots,K, \end{aligned}  $$

where *a*_*ij*_ is the linear regulatory coefficient from gene *j* to gene *i*, *b*_*ij*_ is the nonlinear regulatory coefficient from gene *j* to gene *i*, *N* is the total number of genes in the gene network, and *f*(*g*,*μ*) is a nonlinear sigmoid function defined as

2 $$ f(g,\mu) = \frac{1}{1 + e^{-\mu g}},  $$

with *μ* being a parameter to be identified and *I*_0*i*_ being the external bias on the *i*th gene. The noise vector **e**_*k*_=[*e*_*k*,1_,*e*_*k*,2_,…,*e*_*k,N*_]^*T*^ is Gausssian distributed with zero mean and covariance matrix $\mathbf {Q}^{'}_{k}$, for *k*=1,…,*K*.

The goal of inference is to estimate the parameters (coefficients) of the model in (1), which form the basis of the GRN. To that end, the state vector is concatenated with the model parameters to form augmented state vector as follows. Denote **A**=[ *a*_11_,…,*a*_1*N*_,*a*_21_,…,*a*_2*N*_,…,*a*_*N*1_,…,*a*_*NN*_]^*T*^,**B**=[ *b*_11_,…,*b*_1*N*_,*b*_21_,…,*b*_2*N*_,…,*b*_*N*1_,…,*b*_*NN*_]^*T*^,***μ***=[ *μ*_1_,…,*μ*_*N*_]^*T*^ and *I*_0_=[ *I*_01_,…,*I*_0*N*_]^*T*^ and we denote the expression level for all genes at time step *k* by **g**_*k*_=[*g*_*i,k*_,…,*g*_*N,k*_]^*T*^. Then, the augmented state vector can be described by

3 $$ \mathrm{x}_{k} \triangleq \left[\mathbf{g}^{T}_{k},\mathbf{A}^{T},\mathbf{B}^{T},\boldsymbol{\mu}^{T},\mathbf{I}^{T}_{0}\right]^{T} \in \mathbb{R}^{(2N^{2}+3N)}.  $$

The augmented version of the state transition equations include (1) and the following

4 $$ \begin{aligned} a_{ij,k} &= a_{ij,k-1},~~ b_{ij,k} = b_{ij,k-1},\\ \mu_{i,k} &= \mu_{i,k-1}, ~~I_{0i,k} = I_{0i,k-1}, i,~~j=1,\ldots,N.\\ \end{aligned}  $$

Succinctly, the state transition of the dynamic model is written as

5 $$ \mathrm{x}_{k} = f(\mathrm{x}_{k-1}) + \mathrm{w}_{k-1},  $$

where *f*(·) is the nonlinear function associated with (2) and (4); w_*k*_ = [ *e*_*k*,1_,…,*e*_*k,N*_,0,…,0] is the augmented noise vector with covariance matrix $\mathbf {Q}_{k} = \text {diag} ([\mathbf {\!Q}_{k}^{'}~~\mathbf {0}_{2N + 2N^{2}}])$, where **0**_*m*_ denotes an *m*×*m* all-zero matrix.

The measured gene expression levels can be modeled as

6 $$ \mathrm{z}_{k} = \mathit{h}(\mathrm{x}_{k}) + \mathrm{v}_{k},  $$

where z_*k*_ is the output data from the experiments at time *k*, *h*(x_*k*_)=**g**_*k*_ and $\mathrm {v}_{k} \in \mathbb {R}^{N}$ is Gaussian distributed noise with zero mean and covariance matrix $\mathbf {R}_{k} \in \mathbb {R}^{N\times N}$.

Now, we consider the case that some measurement outputs z_*k*_, are missing and the estimation is made from the available measurements, y_*k*_. We assume that z_1_ is available. At time *k*=2, if the measurement output is missing, estimation is done with z_1_ and at any time instant *k*≥3, maximum of two consecutive time points may be missing. In summary, if z_*k*_ is missing estimation is done with z_*k*−1_ and if z_*k*−1_ is unavailable, estimation is done with z_*k*−2_. Thus, the measurement output at each time can be modeled as [27, 37]

7 $$ \mathrm{y}_{k} = \sum_{d=0}^{\min(k-1,2)}{\gamma_{k}^{d}}\mathrm{z}_{k-d} \quad (k\geqslant1)  $$

with

8 $$ {\gamma_{k}^{0}} = 1-\varsigma_{k}, ~ {\gamma_{k}^{1}} = \varsigma_{k}(1-\varsigma_{k-1}),~~\text{and}~~ {\gamma_{k}^{2}} = \varsigma_{k}\varsigma_{k-1},\\  $$

where *ς*_1_=0, *ς*_*k*_ is a Bernoulli random variable with probability $p(\varsigma _{k} = 1) (k\geqslant 2) = q$. Moreover, it is assumed that x_0_,{w_*k*_,*k*≥0}, {v_*k*_,*k*≥1}, {*ς*_*k*_,*k*≥2} are mutually independent. Denote ${p_{k}^{d}}(d = 0,1,2)$ as the probabilities that measurements z_*k*_, z_*k*−1_, and z_*k*−2_ are used at time *k*. Then, we have

9 $$ \begin{aligned} {p_{k}^{0}} & \triangleq p({\gamma_{k}^{0}} = 1) = \mathbb{E}[{\!\gamma_{k}^{0}}] = 1-q, \\ {p_{k}^{1}} & \triangleq p({\gamma_{k}^{1}} = 1) = \mathbb{E}[{\!\gamma_{k}^{1}}] = q(1-q), \\ {p_{k}^{2}} & \triangleq p({\gamma_{k}^{2}} = 1) = \mathbb{E}[\!{\gamma_{k}^{2}}] = q^{2}, \\ \end{aligned}  $$

Finally, (5)–(8) describe the dynamic model we propose for inferring GRNs with one-step or two-step missing measurements.

To estimate the GRN based on (5)–(8), we solve the optimal filtering problem by finding the estimator $\mathbb {E}\left [ \mathrm {x}_{k}|\mathrm {Y}_{k}\right ] $, where $\mathrm {Y}_{k} \triangleq (\mathrm {y}_{1},\ldots, \mathrm {y}_{k})$. With the Bayes rule, the conditional probability density function (PDF) *p*(x_*k*_|Y_*k*_), and subsequently its first two moments, i.e., $\hat {\mathrm {x}}_{k|k} = \mathbb {E}\left [ \mathrm {x}_{k}|\mathrm {Y}_{k}\right ]$ and $\mathrm {P}_{k|k}^{\mathrm {x}\mathrm {x}}=\mathbb {E}\left [ \tilde {\mathrm {x}}_{k|k}\tilde {\mathrm {x}}_{k|k}^{T}|\mathrm {Y}_{k}\right ]$, are recursively obtained through estimating the posterior predictive PDF of the state *p*(x_*k*_|Y_*k*−1_) and the measurement *p*(y_*k*_|Y_*k*−1_), where $\tilde {\mathrm {x}}= \mathrm {x}-\hat {\mathrm {x}}$ is the estimation error. For the purpose of filtering, we will make use of the following Gaussian assumptions:

1. The one-step posterior predictive PDF of the state x_*k*_ conditioned on Y_*k*−1_ is Gaussian, i.e.,
  10 $$ p(\mathrm{x}_{k}|\mathrm{Y}_{k-1}) = \mathcal{N}(\mathrm{x}_{k};\hat{\mathrm{x}}_{k|k-1},\mathrm{P}_{k|k-1}^{\mathrm{x}\mathrm{x}}),  $$
  where
  11 $${} \hat{\mathrm{x}}_{k|k-1} = \mathbb{E}\left[ \mathrm{x}_{k}|\mathrm{Y}_{k-1}\right], ~~ \mathrm{P}_{k|k-1}^{\mathrm{x}\mathrm{x}} = \mathbb{E}\left[ \tilde{\mathrm{x}}_{k|k-1}\tilde{\mathrm{x}}_{k|k-1}^{T}|\mathrm{Y}_{k-1}\right].  $$
2. The one-step posterior predictive PDF of y_*k*_ conditioned on Y_*k*−1_ is Gaussian, i.e.,
  12 $$ p(\mathrm{y}_{k}|\mathrm{Y}_{k-1}) = \mathcal{N}(\mathrm{y}_{k};\hat{\mathrm{y}}_{k|k-1},\mathrm{P}_{k|k-1}^{\mathrm{y}\mathrm{y}}),  $$
  where
  13 $${} \hat{\mathrm{y}}_{k|k-1} = \mathbb{E}\left[ \mathrm{y}_{k}|\mathrm{Y}_{k-1}\right],~~ \mathrm{P}_{k|k-1}^{\mathrm{y}\mathrm{y}} = \mathbb{E}\left[ \tilde{\mathrm{y}}_{k|k-1}\tilde{\mathrm{y}}_{k|k-1}^{T}|\mathrm{Y}_{k-1}\right].  $$

### Gaussian approximation filters with missing measurements

In this section, we briefly present the general GA filtering framework for the PBGA filters with one-step or two-step missing measurements for the state-space dynamic model. In Additional file 1, we detail its derivation, we review different numerical techniques for approximating multidimensional Gaussian weighted integrals that involve nonlinear transformation of random vectors, and we present the algorithm that implements the UKF version of the filter. Given all the measurements up to the present time in the system described in (5) and (6), the standard Gaussian filter operates by updating only the posterior PDF of the state, i.e., *p*(x_*k*_|Y_*k*_) [38]. However, in the case that the measurements are randomly delayed (or missing) by one or two sampling times as described in (7), apart from *p*(x_*k*_|Y_*k*_), the posterior PDFs *p*(v_*k*_|Y_*k*_), *p*(x_*k*−1_|Y_*k*_), and *p*(v_*k*−1_|Y_*k*_) also must be updated. Specifically, substituting (6) and (8) into (7), we obtain

14 $$ \mathrm{y}_{k} = \sum_{d=0}^{2}{\gamma_{k}^{d}}[h(\mathrm{x}_{k-d})+ \mathrm{v}_{k-d}] \quad (k\geqslant3).  $$

Substituting (14) into (13) to incorporate the delayed measurement in the GA filter, whereby $\hat {\mathrm {y}}_{k \vert k-1}$ and P*k*|*k*−1yy depend on the estimates $\hat {\mathrm {x}}_{k-d}$ and $\hat {\mathrm {v}}_{k-d}$, *d*=0,1,2. By the Gaussian assumptions, it boils down to computing the first two moments of *p*(v_*k*−1_|Y_*k*−1_), *p*(x_*k*−2_|Y_*k*−1_), and *p*(v_*k*−2_|Y_*k*−1_). This is achieved through augmenting the state x_*k*_ as follows:

15 $$ \mathrm{x}^{a}_{k} =\left[ \begin{array}{c} \mathrm{x}_{k} \\ \mathrm{v}_{k} \end{array}\right], ~~ \mathrm{\mathfrak{X}}_{k} =\left[ \begin{array}{c} \mathrm{x}_{k-1}^{a} \\ \mathrm{x}_{k}^{a} \end{array}\right].\\  $$

Given the Gaussian approximations to *p*(x_*k*_|Y_*k*_), *p*(v_*k*_|Y_*k*_), *p*(x_*k*−1_|Y_*k*_), and *p*(v_*k*−1_|Y_*k*_), the posterior PDFs $p(\mathrm {x}_{k-1}^{a}|\mathrm {Y}_{k})$, $p(\mathrm {x}_{k}^{a}|\mathrm {Y}_{k})$, and $p(\mathfrak {X}_{k}|\mathrm {Y}_{k})$ of the augmented states $\mathrm {x}_{k-1}^{a}$, $\mathrm {x}_{k}^{a}$, and $\mathfrak {X}_{k}$ are approximated as Gaussian respectively as

16 $$ \begin{aligned} p(\mathrm{x}_{k-1}^{a}|\mathrm{Y}_{k}) &= \mathcal{N}(\mathrm{x}_{k-1}^{a};\hat{\mathrm{x}}_{k-1|k}^{a},\mathrm{P}_{k-1|k}^{aa}), \\ p(\mathrm{x}_{k}^{a}|\mathrm{Y}_{k}) &= \mathcal{N}(\mathrm{x}_{k}^{a};\hat{\mathrm{x}}_{k|k}^{a},\mathrm{P}_{k|k}^{aa}), \\ p(\mathfrak{X}_{k}|\mathrm{Y}_{k}) &= \mathcal{N}(\mathfrak{X}_{k};\hat{\mathfrak{X}}_{k|k},\mathrm{P}_{k|k}^{\mathfrak{X}\mathfrak{X}}), \end{aligned}  $$

where

17 $${} \begin{aligned} \hat{\mathrm{x}}^{a}_{k-1|k} &=\left[ \begin{array}{ll} \hat{\mathrm{x}}_{k-1|k} \\ \hat{\mathrm{v}}_{k-1|k} \end{array}\right],~\mathrm{P}_{k-1|k}^{aa} =\left[ \begin{array}{ll} \mathrm{P}_{k-1|k}^{\mathrm{x}\mathrm{x}} & \mathrm{P}_{k-1|k}^{\mathrm{x}\mathrm{v}}\\ (\mathrm{P}_{k-1|k}^{\mathrm{x}\mathrm{v}})^{T} & \mathrm{P}_{k-1|k}^{\mathrm{v}\mathrm{v}} \end{array}\right],\\ \text{with} ~~ \mathrm{P}_{k-1|k}^{\mathrm{x}\mathrm{v}} &= \mathbb{E}[\tilde{\mathrm{x}}_{k-1|k}\tilde{\mathrm{v}}_{k-1|k}^{T}|\mathrm{Y}_{k}], \end{aligned}  $$

18 $$ \begin{aligned} \hat{\mathrm{x}}^{a}_{k|k} & =\left[ \begin{array}{ll} \hat{\mathrm{x}}_{k|k} \\ \hat{\mathrm{v}}_{k|k} \end{array}\right],~ \mathrm{P}_{k|k}^{aa} = \left[\begin{array}{ll} \mathrm{P}_{k|k}^{\mathrm{x}\mathrm{x}} & \mathrm{P}_{k|k}^{\mathrm{x}\mathrm{v}}\\ (\mathrm{P}_{k|k}^{\mathrm{x}\mathrm{v}})^{T} & \mathrm{P}_{k|k}^{\mathrm{v}\mathrm{v}} \end{array}\right],\\ \text{with}~~~ \mathrm{P}_{k|k}^{\mathrm{x}\mathrm{v}} &= \mathbb{E}[\tilde{\mathrm{x}}_{k|k}\tilde{\mathrm{v}}_{k|k}^{T}|\mathrm{Y}_{k}], \end{aligned}  $$

and

19 $$ \begin{aligned} \hat{\mathfrak{X}}_{k|k} = \left[\begin{array}{ll} \hat{\mathrm{x}}_{k-1|k}^{a} \\ \hat{\mathrm{x}}_{k|k}^{a} \end{array}\right],~ \mathrm{P}_{k|k}^{\mathfrak{X}\mathfrak{X}} & = \left[\begin{array}{ll} \mathrm{P}_{k-1|k}^{aa} & \mathrm{P}_{k-1,k|k}^{aa}\\ (\mathrm{P}_{k-1,k|k}^{aa})^{T} & \mathrm{P}_{k|k}^{aa} \end{array}\right],\\ \text{with} ~~~ \mathrm{P}_{k-1,k|k}^{aa} &= \mathbb{E}[\tilde{\mathrm{x}}_{k-1|k}^{a}\tilde{\mathrm{x}}_{k|k}^{aT}|\mathrm{Y}_{k}]. \end{aligned}  $$

As with the general GA filtering, the filtering procedure consists of the *state update* and *measurement update*.

#### State update

Given the augmented state PDF $p(\mathfrak {X}_{k-1}|\mathrm {Y}_{k-1})$ at time *k*−1, with its mean and covariance defined as

20 $${} \begin{aligned} \hat{\mathfrak{X}}_{k-1|k-1} &=\left[ \begin{array}{ll} \hat{\mathrm{x}}_{k-2|k-1}^{a} \\ \hat{\mathrm{x}}_{k-1|k-1}^{a} \end{array}\right], \\ \mathrm{P}_{k-1|k-1}^{\mathfrak{X}\mathfrak{X}} &= \left[\begin{array}{ll} \mathrm{P}_{k-2|k-1}^{aa} & \mathrm{P}_{k-2,k-1|k-1}^{aa}\\ (\mathrm{P}_{k-2,k-1|k-1}^{aa})^{T} & \mathrm{P}_{k-1|k-1}^{aa} \end{array}\right], \\ \text{with} ~ \mathrm{P}_{k-2,k-1|k-1}^{aa} &= \mathbb{E}\left[\tilde{\mathrm{x}}_{k-2|k-1}^{a}\tilde{\mathrm{x}}_{k-1|k-1}^{aT}|\mathrm{Y}_{k-1}\right], \end{aligned}  $$

the predicted conditional PDF is $p(\mathfrak {X}_{k}|\mathrm {Y}_{k-1}) = \mathcal {N}(\mathfrak {X}_{k};\hat {\mathfrak {X}}_{k|k-1},\mathrm {P}_{k|k-1}^{\mathfrak {X}\mathfrak {X}})$, with

21 $${} \begin{aligned} \hat{\mathfrak{X}}_{k|k-1} &=\left[ \begin{array}{ll} \hat{\mathrm{x}}_{k-1|k-1}^{a} \\ \hat{\mathrm{x}}_{k|k-1} \\ 0_{N \times 1} \end{array}\right] \\ \mathrm{P}_{k|k-1}^{\mathfrak{X}\mathfrak{X}} &=\left[ \begin{array}{lll} \mathrm{P}_{k-1|k-1}^{aa} & \mathrm{P}_{k-1,k|k-1}^{a\mathrm{x}} & 0_{(2N^{2}+4N) \times N} \\ (\mathrm{P}_{k-1,k|k-1}^{a\mathrm{x}})^{T} & \mathrm{P}_{k|k-1}^{\mathrm{x}\mathrm{x}} & 0_{(2N^{2}+3N) \times N}\\ 0_{N \times (2N^{2}+4N)} & 0_{N \times (2N^{2}+3N)} & \mathbf{R}_{k} \end{array}\right] \end{aligned}  $$

where $\hat {\mathrm {x}}^{a}_{k-1|k-1}$ and $\mathrm {P}^{aa}_{k-1|k-1}$ in (21) are available from $\hat {\mathfrak {X}}_{k-1|k-1}$ and $\mathrm {P}^{\mathfrak {X}\mathfrak {X}}_{k-1|k-1}$ in (20), and

22 $${} \begin{aligned} \hat{\mathrm{x}}_{k|k-1} &= \mathbb{E}_{g} \lbrace f(\mathrm{x}_{k-1})|\hat{\mathfrak{X}}_{k-1|k-1}, \mathrm{P}_{k-1|k-1}^{\mathfrak{X}\mathfrak{X}} \rbrace, \\ \mathrm{P}_{k|k-1}^{\mathrm{x}\mathrm{x}} & = \mathbb{E}_{g} \lbrace f(\mathrm{x}_{k-1})f_{k-1}^{T}(\mathrm{x}_{k-1})|\hat{\mathfrak{X}}_{k-1|k-1}, \mathrm{P}_{k-1|k-1}^{\mathfrak{X}\mathfrak{X}}\rbrace \\ & -\hat{\mathrm{x}}_{k|k-1}\hat{\mathrm{x}}_{k|k-1}^{T} + \mathbf{Q}_{k-1}, \\ \mathrm{P}_{k-1,k|k-1}^{a\mathrm{x}} &= \mathbb{E}_{g} \lbrace \mathrm{x}_{k-1}^{a}f_{k-1}^{T}(\mathrm{x}_{k-1}) | \hat{\mathfrak{X}}_{k-1|k-1}, \mathrm{P}_{k-1|k-1}^{\mathfrak{X}\mathfrak{X}} \rbrace\\ &-\hat{\mathrm{x}}_{k-1|k-1}^{a}\hat{\mathrm{x}}_{k|k-1}^{T}. \end{aligned}  $$

For the detailed derivations, see Additional file 1.

#### Measurement update

After obtaining the approximation to the predictive PDF $p(\mathfrak {X}_{k}|\mathrm {Y}_{k-1})$, the Gaussian approximation of the augmented state posterior PDF $p(\mathfrak {X}_{k}|\mathrm {Y}_{k})$ is obtained by the Kalman filter equations:

23 $$ \begin{aligned} \hat{\mathfrak{X}}_{k|k} &= \hat{\mathfrak{X}}_{k|k-1} + \mathrm{K}^{\mathfrak{X}}_{k}(\mathrm{y}_{k} - \hat{\mathrm{y}}_{k|k-1}),\\ \mathrm{P}^{\mathfrak{X}\mathfrak{X}}_{k|k} &= \mathrm{P}^{\mathfrak{X}\mathfrak{X}}_{k|k-1} - \mathrm{K}^{\mathfrak{X}}_{k}\mathrm{P}_{k|k-1}^{\mathrm{y}\mathrm{y}}(\mathrm{K}^{\mathfrak{X}}_{k})^{T},\\ \mathrm{K}^{\mathfrak{X}}_{k} &= \mathrm{P}^{\mathfrak{X}\mathrm{y}}_{k|k-1}(\mathrm{P}_{k|k-1}^{\mathrm{y}\mathrm{y}})^{-1}, \end{aligned}  $$

where $\mathrm {K}_{k}^{\mathfrak {X}}$ is the Kalman gain and

24 $$ \begin{aligned} \hat{\mathrm{y}}_{k|k-1} & = \sum_{d=0}^{\min(k-1,2)}{p_{k}^{d}}\hat{\mathrm{z}}_{k-d|k-1}, \\ \mathrm{P}^{\mathrm{y}\mathrm{y}}_{k|k-1} & = \sum_{d=0}^{\min(k-1,2)}{p_{k}^{d}}\mathrm{P}_{k-d|k-1}^{\mathrm{z}\mathrm{z}} + \\ &\sum_{d=0}^{\min(k-1,2)}({p_{k}^{d}} \hat{\mathrm{z}}_{k-d|k-1}\hat{\mathrm{z}}_{k-d|k-1}^{T}-\hat{\mathrm{y}}_{k|k-1}\hat{\mathrm{y}}_{k|k-1}^{T}), \\ \mathrm{P}_{k|k-1}^{\mathfrak{X}\mathrm{y}} & = \sum_{d=0}^{\min(k-1,2)}{p_{k}^{d}}\mathrm{P}_{k,k-d|k-1}^{\mathfrak{X}\mathrm{z}}. \end{aligned}  $$

The delayed/missing measurement statistics $\hat {\mathrm {z}}_{k-d|k-1}$, $\mathrm {P}_{k-d|k-1}^{\mathrm {z}\mathrm {z}}$, and $\mathrm {P}_{k,k-d|k-1}^{\mathfrak {X}\mathrm {z}}$ are defined as follows.

For *d*=0:

25 $${} {\begin{aligned} \hat{\mathrm{z}}_{k|k-1} & =\! \mathbb{E}_{g} \lbrace h(\mathrm{x}_{k}) \vert \hat{\mathfrak{X}}_{k|k-1},\mathrm{P}_{k|k-1}^{\mathfrak{X}\mathfrak{X}}\rbrace, \\ \mathrm{P}_{k|k-1}^{\mathrm{z}\mathrm{z}} &\,=\, \mathbb{E}_{g} \lbrace h(\mathrm{x}_{k}){h_{k}^{T}}(\mathrm{x}_{k}) \vert\hat{\mathfrak{X}}_{k|k-1}, \mathrm{P}_{k|k-1}^{\mathfrak{X}\mathfrak{X}}\rbrace\!-\hat{\mathrm{z}}_{k|k-1}\hat{\mathrm{z}}_{k|k-1}^{T} \!+ \mathbf{R}_{k}, \\ \mathrm{P}_{k|k-1}^{\mathfrak{X}\mathrm{z}} &\,=\, \mathbb{E}_{g} \lbrace \mathfrak{X}_{k}(h_{k}(\mathrm{x}_{k}) \!+ \mathrm{v}_{k})^{T} \vert \hat{\mathfrak{X}}_{k|k-1}, \mathrm{P}_{k|k-1}^{\mathfrak{X}\mathfrak{X}}\rbrace \,-\,\hat{\mathfrak{X}}_{k|k-1}\hat{\mathrm{z}}_{k|k-1}^{T}, \end{aligned}}  $$

for *d*=1:

26 $$ \begin{aligned} \hat{\mathrm{z}}_{k-1|k-1} & = \mathbb{E}_{g} \lbrace h(\mathrm{x}_{k-1} + \mathrm{v}_{k-1}) \vert \hat{\mathfrak{X}}_{k|k-1}, \mathrm{P}_{k|k-1}^{\mathfrak{X}\mathfrak{X}}\rbrace, \\ \mathrm{P}_{k-1|k-1}^{\mathrm{z}\mathrm{z}} &= \mathbb{E}_{g} \lbrace (h(\mathrm{x}_{k-1}) + \mathrm{v}_{k-1})(h(\mathrm{x}_{k-1}) + \mathrm{v}_{k-1})^{T} \vert \\ & \hspace{7mm} \hat{\mathfrak{X}}_{k|k-1}, \mathrm{P}_{k|k-1}^{\mathfrak{X}\mathfrak{X}}\rbrace-\hat{\mathrm{z}}_{k-1|k-1}\hat{\mathrm{z}}_{k-1|k-1}^{T}, \\ \mathrm{P}_{k,k-1|k-1}^{\mathfrak{X}\mathrm{z}} &= \mathbb{E}_{g} \lbrace \mathfrak{X}_{k}(h(\mathrm{x}_{k-1}) + \mathrm{v}_{k-1})^{T} \vert \hat{\mathfrak{X}}_{k|k-1}, \mathrm{P}_{k|k-1}^{\mathfrak{X}\mathfrak{X}}\rbrace \\ & \hspace{7mm} -\hat{\mathfrak{X}}_{k|k-1}\hat{\mathrm{z}}_{k|k-1}^{T}, \end{aligned}  $$

and for *d*=2:

27 $${} \begin{aligned} \hat{\mathrm{z}}_{k-2|k-1} & = \mathbb{E}_{g} \lbrace h(\mathrm{x}_{k-2} + \mathrm{v}_{k-2}) \vert \hat{\mathfrak{X}}_{k|k-1}, \mathrm{P}_{k|k-1}^{\mathfrak{X}\mathfrak{X}}\rbrace, \\ \mathrm{P}_{k-2|k-1}^{\mathrm{z}\mathrm{z}} &= \mathbb{E}_{g} \lbrace (h(\mathrm{x}_{k-2} + \mathrm{v}_{k-2})(h(\mathrm{x}_{k-2}) + \mathrm{v}_{k-2})^{T} \vert \\ & \hspace{8mm} \hat{\mathfrak{X}}_{k|k-1}, \mathrm{P}_{k|k-1}^{\mathfrak{X}\mathfrak{X}}\rbrace-\hat{\mathrm{z}}_{k-2|k-1}\hat{\mathrm{z}}_{k-2|k-1}^{T}, \\ \mathrm{P}_{k,k-2|k-1}^{\mathfrak{X}\mathrm{z}} &=\left[ \begin{array}{ll} \mathbb{E}_{g} \lbrace \mathrm{x}_{k-1}^{a}\mathrm{z}_{k-2}^{T}|\mathrm{Y}_{k-1} \vert \hat{\mathfrak{X}}_{k-1|k-1},\mathrm{P}_{k-1|k-1}^{\mathfrak{X}\mathfrak{X}} \rbrace \\ \mathbb{E}_{g} \lbrace \mathrm{x}_{k}\mathrm{z}_{k-2}^{T}|\mathrm{Y}_{k-1} \vert \hat{\mathfrak{X}}_{k-1|k-1},\mathrm{P}_{k-1|k-1}^{\mathfrak{X}\mathfrak{X}} \rbrace \\ 0_{N\times N} \end{array}\right] \\ & \hspace{7mm} - \hat{\mathfrak{X}}_{k|k-1} \hat{\mathrm{z}}^{T}_{k-2|k-1}. \end{aligned}  $$

The filtering estimate $\hat {\mathrm {x}}_{k|k}$ and covariance P*k*|*k*xx of the system state are obtained from $\hat {\mathfrak {X}}_{k|k}$ and $\mathrm {P}^{\mathfrak {X}\mathfrak {X}}_{k|k}$ respectively. (See Additional file 1 for derivations).

However, the Gaussian weighted integrals in (22) and (25)–(27) contain nonlinear functions which render the analytical calculation infeasible and the algorithm becomes intractable. To deal with this, we employ the point-based numerical integration techniques, which is presented in Additional file 1.

## Results

We assess the proposed algorithm using both synthetic data and real data. Gold standards or the ground-truths are provided for both categories of data and the inferred networks are “benchmarked" against the gold standards. Benchmarking is done by counting the number of links correctly predicted by the algorithm (true positives, TP), the number of incorrectly predicted links (false positives, FP), the number of true links missed in the inferred network (false negatives, FN), and the number of correctly identified non-existing links (true negatives, TN). Thus, the following performance metrics will be defined accordingly: true positive rate or recall also known as the sensitivity (TPR = TP/(TP+FN)), positive predictive value or precision (PPV = TP/(TP+FP)), and false positive rate (FPR = FP/(FP+TN), where specificity = 1-FPR). All the metrics are computed for different thresholds and the area under the receiver operating characteristic (AUROC) curve and the area under the precision-recall (AUPR) curve are estimated to illustrate the overall inference performance of the algorithms. As the inference result comprises of the estimates of both the linear and nonlinear regulatory coefficients among the genes, if at least one of the regulatory coefficients between any two genes is recovered, the link is designated as TP.

In addition, y_1_=z_1_; at time *k*=2 the measurement output can be missing by one-step; and at any time instant *k*≥3 it can be missing by one-step or two-step. With the prior knowledge of the number of missing data points to be replaced in the experimental output, an estimate of the value of *q*, the success probability of the Bernoulli variable *ς*_*k*_ can be made. Specifically, if the number of missing data points is less than 20% of the total number of data points, a *q* value chosen in the interval [0.05, 0.2] is a good choice. In our experiments, *q*=0.1, so that the probability that z_*k*_ is used in the estimation is ${p^{0}_{k}} = 0.9$, the probability that z_*k*−1_ is used in the estimation ${p^{1}_{k}} = 0.09$, and the probability that z_*k*−2_ is used in the estimation is ${p^{2}_{k}} = 0.01$. In the remainder of this paper, we denote the datasets that have no missing values as the complete measurements (CM) and we demote the datasets with missing but replaced data points as the missing measurements (MM). The MM is created in the following manner: at time *k*, if z_*k*_ is missing and z_*k*−1_ is available, we replace z_*k*_ with z_*k*−1_; otherwise, we replace z_*k*_ with z_*k*−2_, as there can be maximum of two consecutive missing data points in the measurement.

### Synthetic network

**Fig. 1:**
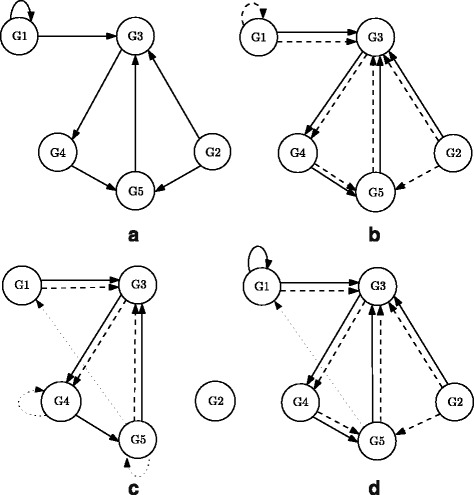
Synthetic network. *Solid black edges* denote the linear connections, *dashed blue edges* denote the nonlinear connections, and the *dotted red arrows* indicate false positives. **a** Gold standard for the synthetic network. **b** Inferred linear and nonlinear connections by the UKF with CM. **c** Inferred linear and nonlinear connections by the UKF with MM. **d** Inferred linear and nonlinear connections with the proposed UKFRMM with MM

The synthetic network in Fig. 1 a is assumed to have both linear and nonlinear connections. The dynamics of the network are based on the model given by (5)–(8), with arrows denoting the direction of regulatory interactions. The parameters of the network, i.e., the linear connection coefficients (LCC) and the nonlinear connection coefficients (NCC), are given in the second column in Table 1 with the NCC in parentheses. The underlying zero-mean Gaussian process noise has a covariance matrix **Q**_*k*_=0.004**I**, and the zero-mean Gaussian measurement noise has a covariance matrix **R**_*k*_=0.001**I**, *k*=1,…,*M*. Time series data are generated for a total of *M*=50 time points. To quantify the results more rigorously, we set the noise threshold at 40% of the maximal variation for linear and nonlinear coefficients such that if an inferred link is less than this threshold, it is considered noise and subsequently filtered off. In the end, we come up with sparse networks and the TPR and PPV metrics are calculated for the networks.

**Table 1:** Network parameters for the synthetic network

Edge	LCC and NCC	UKF with CM	UKF with MM	UKFMM with MM
(1,1)	0.5 (0.4)	— (0.5880)	— —	0.7313 —
(3,1)	0.5 (0.4)	0.3837 (0.4391)	0.7357 (0.5043)	0.4079 (0.4223)
(3,2)	0.5 (0.4)	0.7380 (0.4390)	— —	0.7380 (0.4192)
(3,5)	0.5 (0.4)	0.6098 (0.4391)	0.6623 (0.5043)	0.8285 (0.4354)
(4,3)	0.5 (0.4)	0.7257 (0.3059)	0.3953 (0.2123)	0.7256 (0.3235)
(5,2)	0.5 (0.4)	— (0.3837)	— —	— (0.3235)
(5,4)	0.5 (0.4)	0.6677 (0.3839)	0.5813 (0.3706)	0.7850 (0.3464)
**(4,4)**	**—** **—**	**—** **—**	**—** **(0.8417)**	**—** **—**
**(5,1)**	**—** **—**	**—** **—**	**—** **(0.5916)**	**—** **—**
**(5,5)**	**—** **—**	**—** **—**	**—** **(0.3705)**	**—** **—**
**(1,5)**	**—** **—**	**—** **—**	**—** **—**	**0.5722** **—**

Parameters of the synthetic network and the networks inferred by the UKF algorithm with CM, UKF algorithm with MM, and the proposed UKFMM with MM. The bold edges do not exist in the original network. The false negatives are represented by (non-bold) dashes, and false positives are given in bold numbers

First, we supplied the CM data to the UKF algorithm. The inferred model parameters are shown in the third column in Table 1, with the NCC in parentheses. The corresponding network is displayed in Fig. 1 b where the solid edges indicate the inferred linear connections and the dashed edges indicate the inferred nonlinear connections. Next, we create the MM data by removing data points 10, 11, 25, 35, 36, and 40 from the time series data; the removed data points are then replaced accordingly. To investigate the impact of missing data points on the performance of inference algorithms, we supplied the MM data to the UKF algorithm. The inferred model parameters are shown in the fourth column in Table 1 and the network structure is shown in Fig. 1 c. The black dotted arrows indicate the false positives, i.e., incorrectly predicted links. Finally, using the same MM data we tested the proposed UKF with one-step or two-step missing measurements (UKFMM). The inferred model parameters are shown in the fifth column in Table 1 and the inferred network is displayed in Fig. 1 d. It is observed that the missing data points have great impact on the performance of the UKF algorithm; whereas the proposed UKFMM algorithm can deal with the missing data effectively by displaying a robust performance which is in fact at par with the performance of the UKF with CM. To average out the influence of random data deletion, we run the experiment 1000 times, where at each run, we randomly deleted up to five data points, with maximum of two consecutive data points, and replaced the deleted data points in similar manner as described above. For all the runs, we record the TPR and the PPV, and the average TPR and PPV with their standard deviations (shown in parentheses) are shown in Table 2.

**Table 2:** Average TPR and PPV for the synthetic network (standard deviations are shown in parentheses)

	UKF with CM	UKF with MM	UKFMM with MM
TPR	1.00	0.48 (0.035)	0.91 (0.017)
PPV	1.00	0.53 (0.028)	0.86 (0.013)

### DREAM4 in silico gene regulatory networks

In order to assess the performance of GRN inference algorithms, several in silico gene networks have been produced as the benchmarking data sets, specifically, the DREAM in silico gene networks [39–41]. We made use of the 10-gene networks by the DREAM4 challenge to test the efficacy of the proposed algorithm. All networks and data were generated with version 2.0 of GeneNetWeaver (GNW) [42]. In total, there are five separate networks, each with 10 genes, whose topologies were extracted from the known GRNs in *Escherichia coli* and *Saccharomyces cerevisiae*. The time series measurements were generated using parametrized stochastic differential equations (SDEs), with observations uniformly sampled (21 time points, single replicate) under five different perturbations, for a total of 105 observations per gene. The inference is performed by using all the perturbations. Self-interaction/autoregulatory edges were not expected in the predictions and were subsequently removed. Since the number of possible edges in an *N*-gene network without autoregulatory interactions is *N*(*N*−1), the length of a complete list of predictions is 90 edges for a network of size 10 [33, 34].

We first test the UKF algorithm on the five 10-gene network data sets (CM) and the result is shown in column 2 in Table 3. To average out the influence of random data deletion, we ran 1000 experiments where at each run, we created the MM by randomly deleting up to five data points, with maximum of two consecutive data points, and replaced the deleted data points accordingly. For each run, we fed both the UKF and the proposed UKFMM algorithms with the MM and we record the average AUROC and AUPR scores for each of the five networks, where the empirical averages and standard deviations over 1000 experiments are shown in columns three and six, respectively in Table 3. Again, it is seen from Table 3 that the proposed UKFMM algorithm is robust against the missing data conditions where it can infer the network as accurately as the UKF algorithm that uses the CM.

**Table 3:** AUROC and AUPR curves for the DREAM4 networks

	UKF with CM	UKF with MM	GP4GRN with CM	GP4GRN with MM	UKFMM with MM
N1	[0.63] [0.42]	[0.44(0.024)][0.24(0.020)]	[0.66] [0.42]	[0.42(0.027)][0.29(0.021)]	[0.61(0.015)][0.42(0.008)]
N2	[0.67] [0.49]	[0.48(0.018)][0.26(0.017)]	[0.69] [0.44]	[0.44(0.015)][0.28(0.018)]	[0.64(0.013)][0.44(0.011)]
N3	[0.72] [0.50]	[0.45(0.020)][0.30(0.012)]	[0.70] [0.47]	[0.50(0.022)][0.33(0.016)]	[0.72(0.021)][0.53(0.012)]
N4	[0.75] [0.52]	[0.56(0.019)][0.28(0.011)]	[0.62] [0.35]	[0.36(0.031)][0.25(0.027)]	[0.72(0.009)][0.50(0.010)]
N5	[0.81] [0.44]	[0.53(0.021)][0.26(0.019)]	[0.86] [0.65]	[0.55(0.022)][0.40(0.019)]	[0.80(0.012)][0.42(0.014)]

Column 1 shows the network number. In columns 3, 5, and 6, average AUROC and average AUPR are presented in the *square brackets* and the standard deviations are in *parentheses*

We also compared our algorithm against a relevant computational method designed for the GRN network inference, i.e., [43], which is based on the use of Bayesian analysis with ordinary differential equations (ODEs) and non-parametric Gaussian process, an algorithm referred to as GP4GRN. The inference result of GP4GRN with CM is shown in the fourth column in Table 3. Similarly, we tested GP4GRN with the MM where we ran 1000 experiments. At each run, we created the MM by randomly deleting up to five data points with maximum of two consecutive data points and replaced the deleted data points accordingly. The averages and standard deviations of AUROC and AUPR are obtained and the corresponding results are summarized in the fifth column in Table 3. We conclude that the GP4GRN method has comparable performance to the UKF in all data sets, and similarly it is outperformed by the proposed UKFMM algorithm under missing data conditions.

### *Saccharomyces cerevisiae* IRMA network

*Saccharomyces cerevisiae* GAL network in yeast is one of the most prominent model systems due to its importance for the studies of eukaryotic regulation and relatively self-contained nature [44–47]. A synthetic GRN that contains 5 genes has previously been constructed in the budding yeast [35]. In the well studied network, popularly referred to as in vivo reverse engineering and modeling assessment (IRMA) network, each of the genes regulate at least one other gene in the network. Expression within the network is activated in the presence of galactose and then switched to glucose to obtain the switch-off data which represents the expressive samples at 21 time points. The switch-on data consists of 16 sample points and is obtained by growing the cells in a glucose medium and then changing to galactose.

**Fig. 2:**
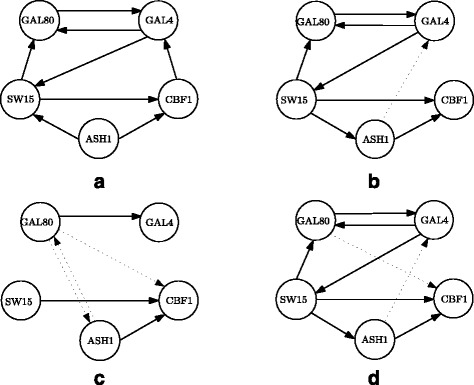
Yeast network. *Solid black edges* denote the combined linear and nonlinear connections, the true positives. The *dotted red edge* is a false positive. **a** Gold standard/ground-truth for the yeast network. **b** Inferred yeast network by the UKF with CM. **c** Inferred yeast network by the UKF with MM. **d** Inferred yeast network by the proposed UKFRMM with MM

The true interactions is shown in Fig. 2 a. The real biological data is first supplied to the UKF algorithm and the inferred network is shown in Fig. 2 b. As standard, some data points are randomly discarded from the input and they are replaced accordingly to generate the MM. The UKF and the proposed algorithm UKFMM are tested on the generated data set (MM) and the inferred networks are shown in Fig. 2 c, d, respectively, and the corresponding results are summarized in Table 4. Again, on the missing data condition, the proposed algorithm shows a better performance compared to the UKF. In addition, we also test the GP4GRN algorithm with both CM and MM and the results are presented in the fourth and fifth columns in Table 4, which further affirms the impact of missing measurements in the GRN inference methods and the relative robustness of the proposed UKFMM algorithm.

**Table 4:** AUROC and AUPR curve for the yeast networks

	UKF with CM	UKF with MM	GP4GRN with CM	GP4GRN with MM	Proposed UKFMM
AUROC curve	0.70	0.42	0.76	0.49	0.68
AUPR curve	0.46	0.34	0.57	0.38	0.46

## Discussion

This work presents a novel algorithm for GRN inference from time-series gene expression data with *one-step* or *two-step missing measurements*. Gene regulation is assumed to follow a nonlinear state evolution model described in (1). The parameters of the model, which are assumed to be the regulatory coefficients between the genes, are estimated with a modified unscented Kalman filtering algorithm. We considered the experimental scenarios that lead to total loss of expression values for all genes at a particular time point or few successive time points which may significantly diminish the performance of GRN inference algorithms.

In the proposed algorithm, the state vector which is the gene expression at each time point in (1) is concatenated with the model parameters and an augmented state vector in (3) is defined for the joint estimation of gene expression values and system parameters. We consider the possibility that each real measurement is randomly missing and the estimation is made from the available measurements. The use of the UKF, an instance of the PBGA filters, for the state and parameter estimation renders the algorithm computationally efficient and capable of working offline or online (when all the measurements are readily available, or they become available successively, respectively). The proposed algorithm is tested on both synthetic and real biological data to evaluate the efficacy of the predictions. From the series of results obtained for both synthetic data and the real biological data, we conclude that the gene network structure can be inferred from time series data with missing values.

In this paper, we have applied the proposed algorithm to the time series data generated from the DNA microarray because to our best of knowledge, DNA microarray is still of interest in transcriptome profiling due to its reduced cost and widespread use as compared to the RNA-seq. In addition, it has been shown that there is there is high correlation between the gene expression profiles generated between the DNA microarray and RNA-seq [48, 49]. Hence, the proposed method can easily be extended to time series gene expression data from RNA-seq.

In general, this work addresses the possibility of having *one-step or two-step missing expression values* by considering them as the delayed observations of the full set of genes. Future work will focus on the inference of the structure of a (potentially larger) network by incorporating a general *s*-step missing values for *s*-consecutive time points, which may address more complex missing data scenarios.

## Conclusions

Time series gene expression data be modeled with state-space model and the model parameters can be estimated using different GA filters. Unfortunately, there are situations which result in loss of expression values for all genes at a particular time point or few successive time points. In this case, conventional filtering approach fails to correctly estimate the model parameters, which are used to elucidate the underlying GRN. We have proposed PBGA filters that treat the missing measurement values as a set of delayed measurements and demonstrated that the modified filter can estimate the model parameters, with missing measurements, as accurate as the conventional filter with no missing measurements.

## Additional file

Additional file 1 Supplemental Material for “Reverse engineering gene regulatory networks from measurement with missing values”. (PDF 207 kb)

## Abbreviations

AUPR: Area under precision-recall
AUROC: Area under the receiver operating characteristic
CKF: Cubature Kalman filter
CDKF: Central difference Kalman filter
CM: Complete measurements
DBN: Dynamic Bayesian network
DNA: Deoxyribonucleic acid
DREAM: Dialogue on reverse engineering assessment and methods
EKF: Extended Kalman filter
FN: False negative
FP: False positive
FPR: False positive rate
GA: Gaussian approximation
GRN: Gene regulatory network
GWN: GeneNetWeaver
IRMA: In-vivo reverse-engineering and modeling assessment
LCC: Linear connection coefficient
MM: Missing measurements
NCC: Nonlinear connection coefficient
NCS: Networked control systems
ODE: Ordinary differential equation
PBGA: Point-based Gaussian approximation
PDF: Probability density function
PPV: Positive predictive value
RNA: Ribonucleic acid
SDE: Stochastic differential equation
TN: True negative
TP: True positive
TPR: True positive rate
UKF: Unscented Kalman filter
UKFMM: Unscented Kalman filter with one-step or two-step missing measurements
UT: Unscented transform
