# Supplementary material for: Reverse engineering gene regulatory networks from measurement with missing values
Source: EURASIP J Bioinform Syst Biol. 2017 Jan 10;2017:2. doi: 10.1186/s13637-016-0055-8 (PMC5225239; doi:10.1186/s13637-016-0055-8)
Supplement: Additional file 1 — Supplemental Material for “Reverse engineering gene regulatory networks from measurement with missing values”. (PDF 207 kb) [file 13637_2016_55_MOESM1_ESM.pdf]

Supplemental Material for "Reverse engineering gene regulatory networks from measurement with missing values"

Oyetunji E Ogundijo [1], Abdulkadir Elmas [1] and Xiaodong Wang [1]

[1] Department of Electrical Engineering,
Columbia University,
New York, NY 10027, USA.

1 GA filter with one-step or two-step missing measurements

We present the derivation of the GA filter equations (state update and measurement update) involved in the estimation of GRN with one-step or two-step missing measurements.

1.1 State Update:

Given $\hat{\mathfrak{X}}_{k-1|k-1}$ and $\mathrm{P}^{\mathfrak{X}\mathfrak{X}}_{k-1|k-1}$ are available at time $k-1$ and are defined as:

$$\begin{aligned} \hat{\mathfrak{X}}_{k-1|k-1} &= \begin{bmatrix} \hat{\mathrm{x}}^a_{k-2|k-1} \\ \hat{\mathrm{x}}^a_{k-1|k-1} \end{bmatrix}, \\ \mathrm{P}^{\mathfrak{X}\mathfrak{X}}_{k-1|k-1} &= \begin{bmatrix} \mathrm{P}^{aa}_{k-2|k-1} & \mathrm{P}^{aa}_{k-2,k-1|k-1} \\ (\mathrm{P}^{aa}_{k-2,k-1|k-1})^T & \mathrm{P}^{aa}_{k-1|k-1} \end{bmatrix}. \end{aligned} \tag{28}$$

We assume that v_k is uncorrelated with Y_{k-1}. Given the Gaussian assumption in (10) - (11), the Gaussian distributions in (16) and the fact that $p(\mathfrak{X}_{k-1}|\mathrm{Y}_{k-1})$ is Gaussian, then the joint PDF of x^a_{k-1}, x_k and v_k conditioned on Y_{k-1} is also Gaussian:

$$p(\mathfrak{X}_k|\mathrm{Y}_{k-1}) = \mathcal{N}(\mathfrak{X}_k; \hat{\mathfrak{X}}_{k|k-1}, \mathrm{P}^{\mathfrak{X}\mathfrak{X}}_{k|k-1}), \tag{29}$$

where

$$\begin{aligned}\hat{\mathfrak{X}}_{k|k-1} &= \mathbb{E}\left[\mathfrak{X}_k|\mathrm{Y}_{k-1}\right] \\ &= \begin{bmatrix}\hat{\mathrm{x}}^a_{k-1|k-1}\\ \hat{\mathrm{x}}_{k|k-1}\\ 0_{N\times 1}\end{bmatrix},\end{aligned} \tag{30}$$

and considering the fact that $\tilde{\mathrm{x}}^a_{k-1|k-1}$, $\tilde{\mathrm{x}}_{k|k-1}$ and Y_{k-1} are uncorrelated with v_k, then:

$$\begin{aligned}\mathrm{P}^{\mathfrak{X}\mathfrak{X}}_{k|k-1} &= \mathbb{E}\{\begin{bmatrix}\tilde{\mathrm{x}}^a_{k-1|k-1}\\ \tilde{\mathrm{x}}_{k|k-1}\\ \mathrm{v}_k\end{bmatrix}[\tilde{\mathrm{x}}^a_{k-1|k-1}\ \tilde{\mathrm{x}}_{k|k-1}\ \mathrm{v}_k|\mathrm{Y}_{k-1}]\} \\ &= \begin{bmatrix}\mathrm{P}^{aa}_{k-1|k-1} & \mathrm{P}^{a\mathrm{x}}_{k-1,k|k-1} & 0_{(2N^2+4N)\times N}\\ (\mathrm{P}^{a\mathrm{x}}_{k-1,k|k-1})^T & \mathrm{P}^{\mathrm{xx}}_{k|k-1} & 0_{(2N^2+3N)\times N}\\ 0_{N\times(2N^2+4N)} & 0_{N\times(2N^2+3N)} & \mathbf{R}_k\end{bmatrix},\end{aligned} \tag{31}$$

where $\hat{\mathrm{x}}^a_{k-1|k-1}$ in (30) and $\mathrm{P}^{aa}_{k-1|k-1}$ in (31) are given in $\hat{\mathfrak{X}}_{k-1|k-1}$ and $\mathrm{P}^{\mathfrak{X}\mathfrak{X}}_{k-1|k-1}$ respectively in (28). However, to obtain $\hat{\mathrm{x}}_{k|k-1}$, $\mathrm{P}^{\mathrm{xx}}_{k|k-1}$ and $\mathrm{P}^{a\mathrm{x}}_{k-1,k|k-1}$ we do the following.

Given that w_{k-1} is uncorrelated with Y_{k-1}, and from (5) and (11), we obtain $\hat{\mathrm{x}}_{k|k-1}$ as follows:

$$\begin{aligned}\hat{\mathrm{x}}_{k|k-1} &= \mathbb{E}\left[\mathrm{x}_k|\mathrm{Y}_{k-1}\right] \\ &= \mathbb{E}\left[f(\mathrm{x}_{k-1}) + \mathrm{w}_{k-1}|\mathrm{Y}_{k-1}\right] \\ &= \mathbb{E}_g\{f(\mathbf{x}_{k-1})|\hat{\mathfrak{X}}_{k-1|k-1}, \mathrm{P}^{\mathfrak{X}\mathfrak{X}}_{k-1|k-1}\},\end{aligned} \tag{32}$$

likewise, $\mathrm{P}^{\mathrm{xx}}_{k|k-1}$ is obtained as follows:

$$\begin{aligned}\mathrm{P}^{\mathrm{xx}}_{k|k-1} &= \mathbb{E}\left[\tilde{\mathrm{x}}_{k|k-1}\tilde{\mathrm{x}}^T_{k|k-1}|\mathrm{Y}_{k-1}\right] \\ &= \mathbb{E}\left[\mathrm{x}_k\mathrm{x}^T_k|\mathrm{Y}_{k-1}\right] - \hat{\mathrm{x}}_{k|k-1}\hat{\mathrm{x}}^T_{k|k-1} \\ &= \mathbb{E}\left[f(\mathrm{x}_{k-1})f^T(\mathrm{x}_{k-1})|\mathrm{Y}_{k-1}\right] \\ &\quad - \hat{\mathrm{x}}_{k|k-1}\hat{\mathrm{x}}^T_{k|k-1} + \mathbf{Q}_{k-1} \\ &= \mathbb{E}_g\{f(\mathrm{x}_{k-1})f^T(\mathrm{x}_{k-1})|\hat{\mathfrak{X}}_{k-1|k-1}, \mathrm{P}^{\mathfrak{X}\mathfrak{X}}_{k-1|k-1}\} \\ &\quad - \hat{\mathrm{x}}_{k|k-1}\hat{\mathrm{x}}^T_{k|k-1} + \mathbf{Q}_{k-1}.\end{aligned} \tag{33}$$

Assuming that w_{k-1} and x^a_{k-1} are uncorrelated, we obtain $\mathrm{P}^{a\mathrm{x}}_{k-1,k|k-1}$ as follows:

$$
\begin{aligned}
\mathrm{P}^{a\mathbf{x}}_{k-1,k|k-1} &= \mathbb{E}\left[\tilde{\mathrm{x}}^a_{k-1|k-1}\tilde{\mathrm{x}}^T_{k|k-1}|\mathrm{Y}_{k-1}\right] \\
&= \mathbb{E}\left[\mathrm{x}^a_{k-1}\mathrm{x}^T_k|\mathrm{Y}_{k-1}\right] - \hat{\mathrm{x}}^a_{k-1|k-1}\hat{\mathrm{x}}^T_{k|k-1} \\
&= \mathbb{E}\left[\mathrm{x}^a_{k-1}(f(\mathrm{x}_{k-1}) + \mathrm{w}_{k-1})^T|\mathrm{Y}_{k-1}\right] \\
&\quad - \hat{\mathrm{x}}^a_{k-1|k-1}\hat{\mathrm{x}}^T_{k|k-1} \\
&= \mathbb{E}_g\{\mathrm{x}^a_{k-1}f^T(\mathrm{x}_{k-1})|\hat{\mathfrak{x}}_{k-1|k-1}, \mathrm{P}^{\mathfrak{x}\mathfrak{x}}_{k-1|k-1}\} \\
&\qquad - \hat{\mathrm{x}}^a_{k-1|k-1}\hat{\mathrm{x}}^T_{k|k-1}.
\end{aligned}
\tag{34}
$$

1.2 Measurement Update:

After we approximate the predictive PDF with $\hat{\mathfrak{x}}_{k|k-1}$ and $\mathrm{P}^{\mathfrak{x}\mathfrak{x}}_{k|k-1}$, the Gaussian approximation of the filtering PDF will be obtained by computing the first two moments of the augmented state $\hat{\mathfrak{x}}_{k|k}$ and $\mathrm{P}^{\mathfrak{x}\mathfrak{x}}_{k|k}$. This is achieved by using the Kalman filter equations:

$$
\begin{aligned}
\hat{\mathfrak{x}}_{k|k} &= \hat{\mathfrak{x}}_{k|k-1} + \mathrm{K}^{\mathfrak{x}}_k(\mathrm{y}_k - \hat{\mathrm{y}}_{k|k-1}), \\
\mathrm{P}^{\mathfrak{x}\mathfrak{x}}_{k|k} &= \mathrm{P}^{\mathfrak{x}\mathfrak{x}}_{k|k-1} - \mathrm{K}^{\mathfrak{x}}_k\mathrm{P}^{\mathrm{yy}}_{k|k-1}(\mathrm{K}^{\mathfrak{x}}_k)^T, \\
\mathrm{K}^{\mathfrak{x}}_k &= \mathrm{P}^{\mathfrak{x}\mathrm{y}}_{k|k-1}(\mathrm{P}^{\mathrm{yy}}_{k|k-1})^{-1},
\end{aligned}
\tag{35}
$$

where $\mathrm{K}^{\mathfrak{x}}_k$ is the Kalman gain. Next, we will derive each of the expressions in (35).

Substituting the delayed/missing measurement function described in (7) - (9) into (13) yields:

$$
\hat{\mathrm{y}}_{k|k-1} = \sum_{d=0}^{\min(k-1,2)} p^d_k\hat{\mathrm{z}}_{k-d|k-1}. \tag{36}
$$

Using the equations (7) and (36), the measurement error (innovation) can be written in terms of the errors in the delayed measurements z_{k-d}:

$$
\begin{aligned}
\tilde{\mathrm{y}}_{k|k-1} &= \mathrm{y}_k - \hat{\mathrm{y}}_{k|k-1} \\
&= \sum_{d=0}^{\min(k-1,2)} \gamma^d_k(\mathrm{z}_{k-d} - \hat{\mathrm{z}}_{k-d|k-1}) - \sum_{d=0}^{\min(k-1,2)} (p^d_k - \gamma^d_k)\hat{\mathrm{z}}_{k-d|k-1}.
\end{aligned}
\tag{37}
$$

Given that γ_k^d is independent of measurement z_k, substituting (8) - (9) and (37) into the definition of $\mathrm{P}^{\mathrm{yy}}_{k|k-1}$ in (13) , we express this conditional covariance as follows:

$$\mathrm{P}^{\mathrm{yy}}_{k|k-1} = \sum_{d=0}^{\min(k-1,2)} p_k^d \mathrm{P}^{\mathrm{zz}}_{k-d|k-1} + \sum_{d=0}^{\min(k-1,2)} (p_k^d \hat{\mathrm{z}}_{k-d|k-1}\hat{\mathrm{z}}^T_{k-d|k-1} - \hat{\mathrm{y}}_{k|k-1}\hat{\mathrm{y}}^T_{k|k-1}), \tag{38}$$

and similarly $\mathrm{P}^{\mathfrak{x}\mathrm{y}}_{k|k-1}$ can be written as:

$$\mathrm{P}^{\mathfrak{x}\mathrm{y}}_{k|k-1} = \sum_{d=0}^{\min(k-1,2)} p_k^d \mathrm{P}^{\mathfrak{x}\mathrm{z}}_{k,k-d|k-1}. \tag{39}$$

Next, we must obtain the expressions $\hat{\mathrm{z}}_{k-d|k-1}$, $\mathrm{P}^{\mathrm{zz}}_{k-d|k-1}$ and $\mathrm{P}^{\mathfrak{x}\mathrm{z}}_{k,k-d|k-1}$ for $d = 0, 1, 2$.

Noting that v_k is zero mean Gaussian noise with covariance $\mathbf{R}_k$ and it is independent of Y_{k-1}, then (for $d = 0$) we obtain $\hat{\mathrm{z}}_{k|k-1}$, $\mathrm{P}^{\mathrm{zz}}_{k|k-1}$ and $\mathrm{P}^{\mathfrak{x}\mathrm{z}}_{k|k-1}$ as follows. By using (6):

$$\begin{aligned} \hat{\mathrm{z}}_{k|k-1} &= \mathbb{E}\left[(h(\mathrm{x}_k) + \mathrm{v}_k)|\mathrm{Y}_{k-1}\right] \\ &= \mathbb{E}_g\{h(\mathrm{x}_k)|\hat{\mathfrak{X}}_{k|k-1}, \mathrm{P}^{\mathfrak{X}\mathfrak{X}}_{k|k-1}\}, \end{aligned} \tag{40}$$

$$\begin{aligned} \mathrm{P}^{\mathrm{zz}}_{k|k-1} &= \mathbb{E}\left[(h(\mathrm{x}_k) + \mathrm{v}_k)(h(\mathrm{x}_k) + \mathrm{v}_k)^T|\mathrm{Y}_{k-1}\right] \\ &\quad - \hat{\mathrm{z}}_{k|k-1}\hat{\mathrm{z}}^T_{k|k-1} \\ &= \mathbb{E}\left[h(\mathrm{x}_k)h^T(\mathrm{x}_k)|\mathrm{Y}_{k-1}\right] + \mathbb{E}\left[\mathrm{v}_k\mathrm{v}_k^T|\mathrm{Y}_{k-1}\right] \\ &\quad - \hat{\mathrm{z}}_{k|k-1}\hat{\mathrm{z}}^T_{k|k-1} \\ &= \mathbb{E}_g\{h(\mathrm{x}_k)h^T(\mathrm{x}_k)|\hat{\mathfrak{X}}_{k|k-1}, \mathrm{P}^{\mathfrak{X}\mathfrak{X}}_{k|k-1}\} - \hat{\mathrm{z}}_{k|k-1}\hat{\mathrm{z}}^T_{k|k-1} + \mathbf{R}_k, \end{aligned} \tag{41}$$

and

$$\begin{aligned} \mathrm{P}^{\mathfrak{X}\mathrm{z}}_{k|k-1} &= \mathbb{E}\left[\mathfrak{X}_k\mathrm{z}_k^T|\mathrm{Y}_{k-1}\right] - \hat{\mathfrak{X}}_{k|k-1}\hat{\mathrm{z}}^T_{k|k-1} \\ &= \mathbb{E}\left[\mathfrak{X}_k(h(\mathrm{x}_k) + \mathrm{v}_k)^T|\mathrm{Y}_{k-1}\right] - \hat{\mathfrak{X}}_{k|k-1}\hat{\mathrm{z}}^T_{k|k-1} \\ &= \mathbb{E}_g\{\mathfrak{X}_k(h(\mathrm{x}_k) + \mathrm{v}_k)^T|\hat{\mathfrak{X}}_{k|k-1}, \mathrm{P}^{\mathfrak{X}\mathfrak{X}}_{k|k-1}\} - \hat{\mathfrak{X}}_{k|k-1}\hat{\mathrm{z}}^T_{k|k-1}. \end{aligned} \tag{42}$$

Similarly, for $d = 1$, $\hat{\mathrm{z}}_{k-1|k-1}$, $\mathrm{P}^{\mathrm{zz}}_{k-1|k-1}$ and $\mathrm{P}^{\mathfrak{X}\mathrm{z}}_{k,k-1|k-1}$ are obtained

$$\begin{aligned} \hat{\mathrm{z}}_{k-1|k-1} &= \mathbb{E}\left[(h(\mathrm{x}_{k-1}) + \mathrm{v}_{k-1})|\mathrm{Y}_{k-1}\right] \\ &= \mathbb{E}_g\{(h(\mathrm{x}_{k-1}) + \mathrm{v}_{k-1})|\hat{\mathfrak{X}}_{k|k-1}, \mathrm{P}^{\mathfrak{X}\mathfrak{X}}_{k,k|k-1}\}, \end{aligned} \tag{43}$$

$$
\begin{aligned}
\mathrm{P}^{\mathrm{zz}}_{k-1|k-1} &= \mathbb{E}\left[(h(\mathrm{x}_{k-1})+\mathrm{v}_{k-1})(h(\mathrm{x}_{k-1})+\mathrm{v}_{k-1})^T|\mathrm{Y}_{k-1}\right] \\
&\quad - \hat{\mathrm{z}}_{k-1|k-1}\hat{\mathrm{z}}^T_{k-1|k-1} \\
&= \mathbb{E}_g\{(h(\mathrm{x}_{k-1})+\mathrm{v}_{k-1})(h(\mathrm{x}_{k-1})+\mathrm{v}_{k-1})^T|\hat{\mathfrak{X}}_{k|k-1},\mathrm{P}^{\mathfrak{X}\mathfrak{X}}_{k|k-1}\} \\
&\quad - \hat{\mathrm{z}}_{k-1|k-1}\hat{\mathrm{z}}^T_{k-1|k-1},
\end{aligned} \tag{44}
$$

and

$$
\begin{aligned}
\mathrm{P}^{\mathfrak{X}\mathrm{z}}_{k,k-1|k-1} &= \mathbb{E}\left[\mathfrak{X}_k \mathrm{z}^T_{k-1}|\mathrm{Y}_{k-1}\right] - \hat{\mathfrak{X}}_{k|k-1}\hat{\mathrm{z}}^T_{k-1|k-1} \\
&= \mathbb{E}\left[\mathfrak{X}_k(h(\mathrm{x}_{k-1})+\mathrm{v}_{k-1})^T|\mathrm{Y}_{k-1}\right] - \hat{\mathfrak{X}}_{k|k-1}\hat{\mathrm{z}}^T_{k-1|k-1} \\
&= \mathbb{E}_g\{\mathfrak{X}_k(h(\mathrm{x}_{k-1})+\mathrm{v}_{k-1})^T|\hat{\mathfrak{X}}_{k|k-1},\mathrm{P}^{\mathfrak{X}\mathfrak{X}}_{k|k-1})\} - \hat{\mathfrak{X}}_{k|k-1}\hat{\mathrm{z}}^T_{k-1|k-1}.
\end{aligned} \tag{45}
$$

Lastly, for $d = 2$, $\hat{\mathrm{z}}_{k-2|k-1}$, $\mathrm{P}^{\mathrm{zz}}_{k-2|k-1}$ and $\mathrm{P}^{\mathfrak{X}\mathrm{z}}_{k,k-2|k-1}$ are obtained as follows:

$$
\begin{aligned}
\hat{\mathrm{z}}_{k-2|k-1} &= \mathbb{E}\left[(h(\mathrm{x}_{k-2})+\mathrm{v}_{k-2})|\mathrm{Y}_{k-1}\right] \\
&= \mathbb{E}_g\{h(\mathrm{x}_{k-2})+\mathrm{v}_{k-2}|\hat{\mathfrak{X}}_{k-1|k-1},\mathrm{P}^{\mathfrak{X}\mathfrak{X}}_{k-1|k-1}\},
\end{aligned} \tag{46}
$$

$$
\begin{aligned}
\mathrm{P}^{\mathrm{zz}}_{k-2|k-1} &= \mathbb{E}\left[(h(\mathrm{x}_{k-2})+\mathrm{v}_{k-2})(h(\mathrm{x}_{k-2})+\mathrm{v}_{k-2})^T|\mathrm{Y}_{k-1}\right] \\
&\quad - \hat{\mathrm{z}}_{k-2|k-1}\hat{\mathrm{z}}^T_{k-2|k-1} \\
&= \mathbb{E}_g\{(h(\mathrm{x}_{k-2})+\mathrm{v}_{k-2})(h(\mathrm{x}_{k-2})+\mathrm{v}_{k-2})^T|\hat{\mathfrak{X}}_{k-1|k-1},\mathrm{P}^{\mathfrak{X}\mathfrak{X}}_{k-1|k-1}\} \\
&\quad - \hat{\mathrm{z}}_{k-2|k-1}\hat{\mathrm{z}}^T_{k-2|k-1},
\end{aligned} \tag{47}
$$

and

$$
\begin{aligned}
\mathrm{P}^{\mathfrak{X}\mathrm{z}}_{k,k-2|k-1} &= \mathbb{E}\{\begin{bmatrix}\tilde{\mathrm{x}}^a_{k-1|k-1}\\ \tilde{\mathrm{x}}_{k|k-1}\\ \mathrm{v}_k\end{bmatrix}\hat{\mathrm{z}}^T_{k-2|k-1}|\mathrm{Y}_{k-1}\} \\
&= \begin{bmatrix}\mathbb{E}[\mathrm{x}^a_{k-1}\mathrm{z}^T_{k-2}|\mathrm{Y}_{k-1}] - \hat{\mathrm{x}}^a_{k-1|k-1}\hat{\mathrm{z}}^T_{k-2|k-1}\\ \mathbb{E}[\mathrm{x}_k\mathrm{z}^T_{k-2}|\mathrm{Y}_{k-1}] - \hat{\mathrm{x}}_{k|k-1}\hat{\mathrm{z}}^T_{k-2|k-1}\\ 0_{N\times N}\end{bmatrix} \\
&= \begin{bmatrix}\mathbb{E}[\mathrm{x}^a_{k-1}\mathrm{z}^T_{k-2}|\mathrm{Y}_{k-1}]\\ \mathbb{E}[\mathrm{x}_k\mathrm{z}^T_{k-2}|\mathrm{Y}_{k-1}]\\ 0_{N\times N}\end{bmatrix} - \hat{\mathfrak{X}}_{k|k-1}\hat{\mathrm{z}}^T_{k-2|k-1},
\end{aligned} \tag{48}
$$

where the expectation terms are given by

$$\begin{aligned}\mathbb{E}\left[\mathrm{x}_{k-1}^{a}\mathrm{z}_{k-2}^{T}|\mathrm{Y}_{k-1}\right] &= \mathbb{E}\left[\mathrm{x}_{k-1}^{a}(h(\mathrm{x}_{k-2})+\mathrm{v}_{k-2})^{T}|\mathrm{Y}_{k-1}\right] \\ &= \mathbb{E}_{g}\{\mathrm{x}_{k-1}^{a}(h(\mathrm{x}_{k-2})+\mathrm{v}_{k-2})^{T}| \\ &\qquad \hat{\mathfrak{X}}_{k-1|k-1},\mathrm{P}_{k-1|k-1}^{\mathfrak{X}\mathfrak{X}}\},\end{aligned} \tag{49}$$

and

$$\mathbb{E}\left[\mathrm{x}_{k}\mathrm{z}_{k-2}^{T}|\mathrm{Y}_{k-1}\right] = \mathbb{E}\left[(f(\mathrm{x}_{k-1})+\mathrm{w}_{k-1})\mathrm{z}_{k-2}^{T}|\mathrm{Y}_{k-1}\right]. \tag{50}$$

Considering that w_{k-1} is uncorrelated with z_{k-2}, (50) can be computed as:

$$\begin{aligned}\mathbb{E}\left[\mathrm{x}_{k}\mathrm{z}_{k-2}^{T}|\mathrm{Y}_{k-1}\right] &= \mathbb{E}\left[f(\mathrm{x}_{k-1})\mathrm{z}_{k-2}^{T}|\mathrm{Y}_{k-1}\right] \\ &= \mathbb{E}\left[f(\mathrm{x}_{k-1})(h(\mathrm{x}_{k-2})+\mathrm{v}_{k-2})^{T}|\mathrm{Y}_{k-1}\right] \\ &= \mathbb{E}_{g}\{f(\mathrm{x}_{k-1})(h(\mathrm{x}_{k-2})+\mathrm{v}_{k-2})^{T}| \\ &\qquad \hat{\mathfrak{X}}_{k-1|k-1},\mathrm{P}_{k-1|k-1}^{\mathfrak{X}\mathfrak{X}}\}.\end{aligned} \tag{51}$$

Finally, given $\hat{\mathfrak{X}}_{k-1|k-1}$ and $\mathrm{P}_{k-1|k-1}^{\mathfrak{X}\mathfrak{X}}$ at time $k-1$, Gaussian approximations of $p(\mathrm{y}_k|\mathrm{Y}_{k-1})$, $p(\mathfrak{X}_k|\mathrm{Y}_{k-1})$ and $p(\mathfrak{X}_k,\mathrm{y}_k|\mathrm{Y}_{k-1})$ are also Gaussian,i.e.,

$$p(\mathfrak{X}_k,\mathrm{y}_k|\mathrm{Y}_{k-1}) = \mathcal{N}(\begin{bmatrix}\mathfrak{X}_k \\ \mathrm{y}_k\end{bmatrix};\begin{bmatrix}\hat{\mathfrak{X}}_{k|k-1} \\ \hat{\mathrm{y}}_{k|k-1}\end{bmatrix},\begin{bmatrix}\mathrm{P}_{k|k-1}^{\mathfrak{X}\mathfrak{X}} & \mathrm{P}_{k|k-1}^{\mathfrak{X}\mathrm{y}} \\ (\mathrm{P}_{k|k-1}^{\mathfrak{X}\mathrm{y}})^{T} & \mathrm{P}_{k|k-1}^{\mathrm{yy}}\end{bmatrix}). \tag{52}$$

With Bayes rule, the GA of $p(\mathfrak{X}_k|\mathrm{Y}_k)$ with filtering estimation $\hat{\mathfrak{X}}_{k|k}$ and the covariance $\mathrm{P}_{k|k}^{\mathfrak{X}\mathfrak{X}}$ at time k of the augmented state is:

$$p(\mathfrak{X}_k|\mathrm{Y}_k) = \frac{p(\mathfrak{X}_k,\mathrm{y}_k|\mathrm{Y}_{k-1})}{p(\mathrm{y}_k|\mathrm{Y}_{k-1})}. \tag{53}$$

The joint PDF is expressed as:

$$p(\mathfrak{X}_k,\mathrm{y}_k|\mathrm{Y}_{k-1}) = \frac{1}{\sqrt{|2\pi\Sigma|}}\exp(-\frac{1}{2}\begin{bmatrix}\tilde{\mathfrak{X}}_{k|k-1}^{T} & \tilde{\mathrm{y}}_{k|k-1}^{T}\end{bmatrix}\Sigma^{-1}\begin{bmatrix}\tilde{\mathfrak{X}}_{k|k-1}^{T} \\ \tilde{\mathrm{y}}_{k|k-1}^{T}\end{bmatrix}) \tag{54}$$

where

$$\Sigma = \begin{bmatrix}\mathrm{P}_{k|k-1}^{\mathfrak{X}\mathfrak{X}} & \mathrm{P}_{k|k-1}^{\mathfrak{X}\mathrm{y}} \\ (\mathrm{P}_{k|k-1}^{\mathfrak{X}\mathrm{y}})^{T} & \mathrm{P}_{k|k-1}^{\mathrm{yy}}\end{bmatrix}, \tag{55}$$

we rewrite Σ as:

$$\Sigma = \begin{bmatrix} I_{2(2N^2+4N)} & \mathrm{K}_k^{\mathfrak{X}} \\ 0_{N\times 2(2N^2+4N} & I_N \end{bmatrix} \begin{bmatrix} \mathrm{P}_{k|k-1}^{\mathfrak{X}\mathfrak{X}} & 0_{2(2N^2+4N)\times N} \\ 0_{N\times 2(2N^2+4N} & \mathrm{P}_{k|k-1}^{\mathrm{yy}} \end{bmatrix} \begin{bmatrix} I_{2(2N^2+4N)} & 0_{2(2N^2+4N)\times N} \\ (\mathrm{K}_k^{\mathfrak{X}})^T & I_N \end{bmatrix}, \tag{56}$$

and

$$|\Sigma| = |\mathrm{P}_{k|k-1}^{\mathfrak{X}\mathfrak{X}}||\mathrm{P}_{k|k-1}^{\mathrm{yy}}|, \tag{57}$$

where $\mathrm{K}_k^{\mathfrak{X}}$ and $\mathrm{P}_{k|k}^{\mathfrak{X}\mathfrak{X}}$ are given in (35). Then Σ^{-1} is obtained as:

$$\Sigma^{-1} = \begin{bmatrix} I_L & 0_{m\times L} \\ \mathrm{K}_k^{\mathfrak{X}} & I_m \end{bmatrix} \begin{bmatrix} (\mathrm{P}_{k|k-1}^{\mathfrak{X}\mathfrak{X}})^{-1} & 0_{L\times m} \\ 0_{m\times L} & (\mathrm{P}_{k|k-1}^{\mathrm{yy}})^{-1} \end{bmatrix} \begin{bmatrix} I_L & -\mathrm{K}_k^{\mathfrak{X}})^T \\ 0_{m\times L} & I_m \end{bmatrix}. \tag{58}$$

Substituting (57) - (58) into (54) and coupled with the results obtained in (36) and (38), (54) becomes:

$$\begin{aligned} p(\mathfrak{X}_k, \mathrm{y}_k|\mathrm{Y}_{k-1}) = & \frac{1}{\sqrt{|2\pi\mathrm{P}_{k|k}^{\mathfrak{X}\mathfrak{X}}||2\pi\mathrm{P}_{k|k-1}^{\mathrm{yy}}|}} \exp\{-\frac{1}{2}(\tilde{\mathfrak{X}}_{k|k-1} - \mathrm{K}_k^{\mathfrak{X}}\tilde{\mathrm{y}}_{k|k-1})^T(\mathrm{P}_{k|k}^{\mathfrak{X}\mathfrak{X}})^{-1} \\ & (\tilde{\mathfrak{X}}_{k|k-1} - \mathrm{K}_k^{\mathfrak{X}}\tilde{\mathrm{y}}_{k|k-1}) - \frac{1}{2}(\tilde{\mathrm{y}}_{k|k-1})^T(\mathrm{P}_{k|k-1}^{\mathrm{yy}})^{-1}\tilde{\mathrm{y}}_{k|k-1}\} \\ = & \mathcal{N}(\mathfrak{X}_k; \hat{\mathfrak{X}}_{k|k}, \mathrm{P}_{k|k}^{\mathfrak{X}\mathfrak{X}})p(\mathrm{y}_k|\mathrm{Y}_{k-1}). \end{aligned} \tag{59}$$

Thus, we obtain the posterior PDF $p(\mathfrak{X}_k|\mathrm{Y}_k)$ of the augmented state by substituting (59) into (53) with the mean $\hat{\mathfrak{X}}_{k|k}$ and covariance $\mathrm{P}_{k|k}^{\mathfrak{X}\mathfrak{X}}$ as in (35).

2 Point based numerical integration

For an l-dimensional random vector $x \sim \mathcal{N}(x; \mu, P)$, the first and second moments (mean and covariance) of x can be captured by using a set of points deterministically, called *sigma-points*. Consider the following nonlinear transformation of x, i.e., $y = h(x)$. The mean and covariance of the random vector y and the cross-covariance between x and y can be estimated by propagating each of the sigma points through the nonlinear function, and these estimates are accurate to the second order (or third order for true Gaussian priors) of the Taylor series expansion of $h(x)$ for *any* nonlinear function [1]. Next, we briefly introduce certain sigma-point based numerical integration techniques in the literature.

Unscented Transformation (UT): In the unscented transform, a set of $2l+1$ *sigma-points* are chosen as

$$\begin{aligned}
&\Gamma_o = \mu, \\
&\Gamma_i = \mu + \left(\sqrt{(l+\lambda)P}\right)_i, && i = 1, ..., l, \\
&\Gamma_i = \mu - \left(\sqrt{(l+\lambda)P}\right)_{i-l}, && i = l+1, ..., 2l,
\end{aligned} \tag{60}$$

and their respective weights as

$$\begin{aligned}
w_o^{(m)} &= \frac{\lambda}{l+\lambda}, \\
w_o^{(c)} &= \frac{\lambda}{l+\lambda} + (1-\alpha^2+\beta), \\
w_i^{(m)} &= w_i^{(c)} = \frac{1}{2(l+\lambda)}, && i = 1, 2, ..., 2l,
\end{aligned} \tag{61}$$

where $\sqrt{P} = \text{chol}(P)$, $(P)_i$ represents the ith column of matrix P, α is a scaling parameter and it is usually a small number, $0 \leqslant \alpha \leqslant 1$, in order to avoid oversampling non-local effects when linearities are strong; β is a parameter that incorporates prior knowledge of random variable x, $\beta = 2$ is optimal for Gaussian distribution [2]; $\lambda = \alpha^2(l+\kappa) - l$ where $\kappa \geqslant 0$, guarantees positive semi-definiteness of the covariance matrix during the recursive filtering. Then the mean and variance of y and the cross-covariance of x and y are approximated as:

$$\begin{aligned}
\hat{y} &\approx \sum_{i=0}^{2l} w_i^{(m)} h(\Gamma_i), \\
\mathrm{P}^y &\approx \sum_{i=0}^{2l} w_i^{(c)} (h(\Gamma_i) - \hat{y})(h(\Gamma_i) - \hat{y})^T, \\
\mathrm{P}^{xy} &\approx \sum_{i=0}^{2l} w_i^{(c)} (\Gamma_i - \mu)(h(\Gamma_i) - \hat{y})^T.
\end{aligned} \tag{62}$$

Third-degree Cubature Rule: The third-degree cubature rule is a special form of the UT [3]. A set of sigma points $\Gamma_i, i = 1, ..., 2l$, are chosen (one point short of UT) as follows:

$$\begin{aligned}
&\Gamma_i = \mu + \left(\sqrt{lP}\right)_i, && i = 1, ..., l, \\
&\Gamma_i = \mu - \left(\sqrt{lP}\right)_{i-l}, && i = l+1, ..., 2l,
\end{aligned} \tag{63}$$

with their respective weights as:

$$w_i^{(m)} = w_i^{(c)} = \frac{1}{2l}, \qquad i = 1, ..., 2l. \tag{64}$$

The mean and variance of y and the cross-covariance of x and y are calculated in the same manner as in UT.

Central Difference Rule: In Stirling's approximation of $h(\cdot)$, the derivatives in the Taylor series are replaced by the central divided differences [4]. Correspondingly, we have $2l+1$ points:

$$\begin{aligned} \Gamma_o &= \mu, \\ \Gamma_i &= \mu + h\left(\sqrt{P}\right)_i, & i = 1, ..., l, \\ \Gamma_i &= \mu - h\left(\sqrt{P}\right)_{i-l}, & i = l+1, ...2l, \end{aligned} \tag{65}$$

with their respective weights as

$$\begin{aligned} w_o^{(m)} &= \frac{\delta^2 - l}{\delta^2}, \quad w_i^{(m)} = \frac{1}{2\delta^2}, \quad i = 1, ..., 2l, \\ w_i^{(c1)} &= \frac{1}{4\delta^2}, \quad w_i^{(c2)} = \frac{\delta^2 - 1}{4\delta^4}, \quad i = 1, ..., l, \end{aligned} \tag{66}$$

where δ is the step size which can be set to $\sqrt{3}$ for Gaussian distribution. Given that $\mathcal{Y}_i = h(\Gamma_i)$, the mean and variance of y and the cross-covariance of x and y are approximated as:

$$\begin{aligned} \hat{y} &\approx \sum_{i=0}^{2l} w_i^{(m)} \mathcal{Y}_i, \\ \mathrm{P}^y &\approx \sum_{i=0}^{l} w_i^{(c1)} (\mathcal{Y}_i - \mathcal{Y}_{i+l})(\mathcal{Y}_i - \mathcal{Y}_{i+l})^T + \\ &\quad \sum_{i=0}^{l} w_i^{(c2)} (\mathcal{Y}_i + \mathcal{Y}_{i+l} - 2\mathcal{Y}_o)(\mathcal{Y}_i + \mathcal{Y}_{i+l} - 2\mathcal{Y}_o)^T, \\ \mathrm{P}^{xy} &\approx \sum_{i=0}^{l} w_i^{(m)} (\Gamma_i - \mu)(\mathcal{Y}_i - \mathcal{Y}_{i+l})^T. \end{aligned} \tag{67}$$

3 Algorithm for UKF with one-step or two-step missing measurements

Let $\hat{\mathrm{x}}_{0|0}$ be the initial state, and $\mathrm{P}^{\mathrm{xx}}_{0|0}$ be the initial state covariance. The filter is initialized in the following manner:

$$\hat{\mathrm{x}}^{a}_{0|0} = \begin{bmatrix} \hat{\mathrm{x}}_{0|0} \\ 0_{N\times 1} \end{bmatrix}, \quad \hat{\mathfrak{X}}_{0|0} = \begin{bmatrix} 0_{(2N^2+4N)\times 1} \\ \hat{\mathrm{x}}^{a}_{0|0} \end{bmatrix}$$

$$\mathrm{P}^{aa}_{0|0} = \begin{bmatrix} \mathrm{P}_{0|0} & 0_{(2N^2+3N)\times N} \\ 0_{N\times(2N^2+3N)} & 0_{N\times N} \end{bmatrix}$$

$$\mathrm{P}^{\mathfrak{X}\mathfrak{X}}_{0|0} = \begin{bmatrix} 0_{2N^2+4N} & 0_{2N^2+4N} \\ 0_{2N^2+4N} & \mathrm{P}^{aa}_{0|0} \end{bmatrix}$$

where N is the number of genes in the network. The algorithm iterates the following steps (state update and measurement update) for $k = 1, 2, ...$

State update Approximation of the conditional mean and covariance of $\mathfrak{X}_k$ given Y_{k-1}, i.e., $\hat{\mathfrak{X}}_{k|k-1}$ and $\mathrm{P}^{\mathfrak{X}\mathfrak{X}}_{k|k-1}$.

1. Given $\hat{\mathfrak{X}}_{k-1|k-1}$ and $\mathrm{P}^{\mathfrak{X}\mathfrak{X}}_{k-1|k-1}$, factorize $\mathrm{P}^{\mathfrak{X}\mathfrak{X}}_{k-1|k-1}$ and construct a set of sigma points, $\left\{\Gamma_{i,k-1|k-1}, i = 0, ..., 2l)\right\}$, $l = 2(2N^2+4N)$, and their respective weights as described in (60) - (61) such that:

$$\Gamma_{i,k-1|k-1} = \begin{bmatrix} \mathrm{x}^{a}_{i,k-2|k-1} \\ \mathrm{x}^{a}_{i,k-1|k-1} \end{bmatrix} = \begin{bmatrix} \mathrm{x}_{i,k-2|k-1} \\ \mathrm{v}_{i,k-2|k-1} \\ \mathrm{x}_{i,k-1|k-1} \\ \mathrm{v}_{i,k-1|k-1} \end{bmatrix}.$$

2. Compute the propagated sigmal points:

$$\chi_{i,k|k-1} = f(\mathrm{x}_{i,k-1|k-1}),$$
$$\mathcal{Z}_{i,k-2|k-1} = h(\mathrm{x}_{i,k-2|k-1}) + \mathrm{v}_{i,k-2|k-1}.$$

3. Compute the statistics $\hat{\mathrm{x}}_{k|k-1}$, $\mathrm{P}^{\mathrm{xx}}_{k|k-1}$ and $\mathrm{P}^{a\mathrm{x}}_{k-1,k|k-1}$:

$$\hat{\mathrm{x}}_{k|k-1} = \sum_{i=0}^{2L} w_i^{(m)} \chi_{i,k|k-1},$$

$$\mathrm{P}^{\mathrm{xx}}_{k|k-1} = \sum_{i=0}^{2L} w_i^{(c)} (\chi_{i,k|k-1} - \hat{\mathrm{x}}_{k|k-1})$$
$$(\chi_{i,k|k-1} - \hat{\mathrm{x}}_{k|k-1})^T + \mathbf{Q}_{k-1},$$

and

$$\mathrm{P}^{a\mathrm{x}}_{k-1,k|k-1} = \sum_{i=0}^{2L} w_i^{(c)} (\mathrm{x}^a_{i,k-1|k-1} - \hat{\mathrm{x}}^a_{k-1|k-1})$$
$$(\chi_{i,k|k-1} - \hat{\mathrm{x}}_{k|k-1})^T.$$

Then

$$\hat{\mathfrak{X}}_{k|k-1} = \begin{bmatrix} \hat{\mathrm{x}}^a_{k-1|k-1} \\ \hat{\mathrm{x}}_{k|k-1} \\ 0_{N\times 1} \end{bmatrix},$$
$$\mathrm{P}^{\mathfrak{X}\mathfrak{X}}_{k|k-1} = \begin{bmatrix} \mathrm{P}^{aa}_{k-1|k-1} & \mathrm{P}^{a\mathrm{x}}_{k-1,k|k-1} & 0_{(2N^2+4N)\times N} \\ (\mathrm{P}^{a\mathrm{x}}_{k-1,k|k-1})^T & \mathrm{P}^{\mathrm{xx}}_{k|k-1} & 0_{N\times(2N^2+3N)} \\ 0_{N\times(2N^2+4N)} & 0_{N\times(2N^2+3N)} & \mathbf{R}_k \end{bmatrix}.$$

Measurement update: Approximation of the conditional mean and covariance of $\mathfrak{X}_k$ given Y_k, i.e., $\hat{\mathfrak{X}}_{k|k}$ and $\mathrm{P}^{\mathfrak{X}\mathfrak{X}}_{k|k}$

4. With $\hat{\mathfrak{X}}_{k|k-1}$ and $\mathrm{P}^{\mathfrak{X}\mathfrak{X}}_{k|k-1}$ given, construct a new set of sigma points, $\{\Gamma_{i,k|k-1}, i = 0, ..., 2l)\}$, $l = 2(2N^2 + 4N)$, and their respective weights as described in (60) - (61) such that:

$$\Gamma_{i,k|k-1} = \begin{bmatrix} \mathrm{x}^a_{i,k-1|k-1} \\ \mathrm{x}^a_{i,k|k-1} \end{bmatrix} = \begin{bmatrix} \mathrm{x}_{i,k-1|k-1} \\ \mathrm{v}_{i,k-1|k-1} \\ \mathrm{x}_{i,k|k-1} \\ \mathrm{v}_{i,k|k-1} \end{bmatrix}.$$

5. Compute the propagated sigma points:

$$\mathcal{Z}_{i,k|k-1} = h(\mathrm{x}_{i,k|k-1}),$$
$$\mathcal{Z}_{i,k-1|k-1} = h(\mathrm{x}_{i,k-1|k-1}) + \mathrm{v}_{i,k-1|k-1}.$$

6. Approximate the statistics of z_{k-2} given Y_{k-1}:

$$\hat{\mathrm{z}}_{k-2|k-1} = \sum_{i=0}^{2l} w_i^{(m)} \mathcal{Z}_{i,k-2|k-1},$$

$$\mathrm{P}^{\mathrm{zz}}_{k-2|k-1} = \sum_{i=0}^{2l} w_i^{(c)} (\mathcal{Z}_{i,k-2|k-1} - \hat{\mathrm{z}}_{k-2|k-1})$$
$$(\mathcal{Z}_{i,k-2|k-1} - \hat{\mathrm{z}}_{k-2|k-1})^T,$$

$$\mathbb{E}\left[\mathrm{x}^a_{k-1}\mathrm{z}^T_{k-2}|\mathrm{Y}_{k-1}\right] = \sum_{i=0}^{2l} w_i^{(c)} (\mathrm{x}^a_{i,k-1|k-1}\mathcal{Z}^T_{i,k-2|k-1}),$$
$$\mathbb{E}\left[\mathrm{x}_{k}\mathrm{z}^T_{k-2}|\mathrm{Y}_{k-1}\right] = \sum_{i=0}^{2l} w_i^{(c)} (\chi_{i,k|k-1}\mathcal{Z}^T_{i,k-2|k-1}),$$

then

$$\mathrm{P}^{\mathfrak{x}\mathrm{z}}_{k,k-2|k-1} = \begin{bmatrix} \mathbb{E}\left[\mathrm{x}^a_{k-1}\mathrm{z}^T_{k-2}|\mathrm{Y}_{k-1}\right] \\ \mathbb{E}\left[\mathrm{x}_{k}\mathrm{z}^T_{k-2}|\mathrm{Y}_{k-1}\right] \\ 0_{N\times(2N^2+3N)} \end{bmatrix} - \hat{\mathfrak{X}}_{k|k-1}\hat{\mathrm{z}}^T_{k-2|k-1}.$$

7. Approximate statistics of z_{k-1} given Y_{k-1}:

$$\hat{\mathrm{z}}_{k-1|k-1} = \sum_{i=0}^{2l} w_i^{(m)} \mathcal{Z}_{i,k-1|k-1},$$

$$\mathrm{P}^{\mathrm{zz}}_{k-1|k-1} = \sum_{i=0}^{2l} w_i^{(c)} (\mathcal{Z}_{i,k-1|k-1} - \hat{\mathrm{z}}_{k-1|k-1})$$
$$(\mathcal{Z}_{i,k-1|k-1} - \hat{\mathrm{z}}_{k-1|k-1})^T,$$

and

$$\mathrm{P}^{\mathfrak{x}\mathrm{z}}_{k,k-1|k-1} = \sum_{i=0}^{2L} w_i^{(c)} (\Gamma_{i,k|k-1} - \hat{\mathfrak{X}}_{k|k-1})$$
$$(\mathcal{Z}_{i,k-1|k-1} - \hat{\mathrm{z}}_{k-1|k-1})^T.$$

8. Approximate the statistics of z_k given Y_{k-1}:

$$\hat{\mathrm{z}}_{k|k-1} = \sum_{i=0}^{2l} w_i^{(m)} \mathcal{Z}_{i,k|k-1},$$

$$\mathrm{P}^{\mathrm{zz}}_{k|k-1} = \sum_{i=0}^{2l} w_i^{(c)} (\mathcal{Z}_{i,k|k-1} - \hat{\mathrm{z}}_{k|k-1})$$
$$(\mathcal{Z}_{i,k|k-1} - \hat{\mathrm{z}}_{k|k-1})^T + \mathbf{R}_k,$$

and

$$\mathrm{P}^{\mathfrak{x}\mathrm{z}}_{k|k-1} = \sum_{i=0}^{2l} w_i^{(c)} (\Gamma_{i,k|k-1} - \hat{\mathfrak{x}}_{k|k-1})$$
$$(\mathcal{Z}_{i,k|k-1} - \hat{\mathrm{z}}_{k|k-1})^T.$$

9. Approximate the statistics of y_k given Y_{k-1}:

$$\hat{\mathrm{y}}_{k|k-1} = \sum_{d=0}^{min(k-1,2)} p_k^d \hat{\mathrm{z}}_{k-d|k-1},$$

$$\mathrm{P}^{\mathrm{yy}}_{k|k-1} = \sum_{d=0}^{\min(k-1,2)} p_k^d \mathrm{P}^{\mathrm{zz}}_{k-d,k-d|k-1} +$$
$$\sum_{d=0}^{\min(k-1,2)} (p_k^d \hat{\mathrm{z}}_{k-d|k-1} \hat{\mathrm{z}}^T_{k-d|k-1} - \hat{\mathrm{y}}_{k|k-1} \hat{\mathrm{y}}^T_{k|k-1}),$$

and

$$\mathrm{P}^{\mathfrak{x}\mathrm{y}}_{k|k-1} = \sum_{d=0}^{\min(k-1,2)} p_k^d \mathrm{P}^{\mathfrak{x}\mathrm{z}}_{k,k-d|k-1}.$$

10. Calculate the Kalman gain and update $\hat{\mathfrak{x}}_{k|k}$ and $\mathrm{P}^{\mathfrak{x}\mathfrak{x}}_{k|k}$:

$$\mathrm{K}^{\mathfrak{x}}_k = \mathrm{P}^{\mathfrak{x}\mathrm{y}}_{k,k|k-1} (\mathrm{P}^{\mathrm{yy}}_{k|k-1})^{-1}$$
$$\hat{\mathfrak{x}}_{k|k} = \hat{\mathfrak{x}}_{k|k-1} + \mathrm{K}^{\mathfrak{x}}_k (\mathrm{y}_k - \hat{\mathrm{y}}_{k|k-1})$$
$$\mathrm{P}^{\mathfrak{x}\mathfrak{x}}_{k|k} = \mathrm{P}^{\mathfrak{x}\mathfrak{x}}_{k|k-1} - \mathrm{K}^{\mathfrak{x}}_k \mathrm{P}^{\mathrm{yy}}_{k|k-1} (\mathrm{K}^{\mathfrak{x}}_k)^T$$

11. Obtain the filtering estimate $\hat{\mathrm{x}}_{k|k}$ and covariance $\mathrm{P}^{\mathrm{xx}}_{k|k}$ of the PDF $p(\mathrm{x}|\mathrm{Y}_k)$ from $\hat{\mathfrak{x}}_{k|k}$ and $\mathrm{P}^{\mathfrak{x}\mathfrak{x}}_{k|k}$, respectively.
12. Return to Step 1.
13. $k \leftarrow k+1$.

References

1. Van Der Merwe, R. (2004) Sigma-point Kalman filters for probabilistic inference in dynamic state-space models. PhD Thesis, Oregon Health and Science University, Portland, USA.

2. Julier,S. et al. (1997) New extension of the Kalman filter to nonlinear systems. *International Society for Optics and Photonics*, 182–193.

3. Jia,B. et al. (2013) High-degree cubature Kalman filter. *Automatica*, **49**, 510–518.

4. NøRgaard,M. et al. (2000) New developments in state estimation for nonlinear systems. *Automatica*, **36**, 1627–1638.
